# Duration of untreated illness in eating disorders: a comprehensive systematic review and meta-regression

**DOI:** 10.1186/s40337-026-01731-6

**Published:** 2026-08-19

**Authors:** Gyselle de Geus, Mike Trott, Alessio Maria Monteleone, Joseph Firth, Dan Siskind, Scott Teasdale, Urska Arnautovska, Ravi Iyer, Brendon Stubbs, Natasha Demidenko, David Martin, Ruxandra Antochi, Jess G. Fiedorowicz, Amelia Austin, Ulrike Schmidt, Tracey Wade, Fernando Fernandez-Aranda, Beate Herpertz-Dahlmann, Hans W. Hoek, Ulrich Voderholzer, David Kolar, Stephan Zipfel, Kathleen M. Pike, Andreas Karwautz, Stephen Wonderlich, Lee Smith, Marco Carfagno, Marco Solmi

**Affiliations:** 1 Ottawa Hospital Research Institute, Ottawa, ON Canada; 2 SCIENCES Lab, Department of Psychiatry, University of Ottawa, 501 Smyth Road, Ottawa, ON Canada; 3 Queensland Centre for Mental Health Research, Brisbane, QLD Australia; 4 Faculty of Health, Medicine and Behavioural Sciences, The University of Queensland, Brisbane, QLD Australia; 5 Addiction and Mental Health Service, Metro South Health, Brisbane, QLD Australia; 6 Department of Psychiatry, University of Campania Luigi Vanvitelli, Naples, Italy; 7 Division of Psychology and Mental Health, University of Manchester, Manchester, UK; 8 School of Clinical Medicine, Nutrition, Exercise and Social Equity Research Group, University of New South Wales, Sydney, NSW Australia; 9 Swinburne University of Technology, Centre for Mental Health and Brain Sciences, Melbourne, VIC Australia; 10 Department of Psychological Medicine, King’s College London, London, UK; 11 Center for Sport Science and University Sports, University of Vienna, Vienna, Austria; 12 The Ottawa Hospital, Mental Health Department, Ottawa, ON Canada; 13 Royal Ottawa Mental Health Centre, Ottawa, ON Canada; 14 Department of Psychiatry, University of Ottawa, Ottawa, ON Canada; 15 Department of Community Health Sciences, University of Calgary, Calgary, AB Canada; 16 Centre for Research in Eating and Weight Disorders (CREW), King’s College London, London, UK; 17 South London and Maudsley NHS Foundation Trust, London, UK; 18 Blackbird Initiative, Flinders University, Adelaide, SA Australia; 19 Ciber Fisiopatología Obesidad y Nutrición (CIBERObn), Instituto de Salud Carlos III, Barcelona, Spain; 20 Psychoneurobiology of Eating and Addictive Behaviors Group, IDIBELL, Barcelona, Spain; 21 Department of Clinical Psychology, Bellvitge University Hospital, IDIBELL, Barcelona, Spain; 22 Department of Clinical Sciences, School of Medicine and Health Sciences, University of Barcelona, Barcelona, Spain; 23 Department of Child and Adolescent Psychiatry, RWTH Aachen University, Aachen, Germany; 24 West German Center for Child and Adolescent Health, University Hospital Cologne, Cologne, Germany; 25 Parnassia Psychiatric Institute, The Hague, The Netherlands; 26 Department of Psychiatry, University Medical Center Groningen, Groningen, The Netherlands; 27 Department of Epidemiology, Columbia University, New York, NY USA; 28 Schoen Clinic Roseneck, Prien am Chiemsee, Germany; 29 Department of Psychiatry and Psychotherapy, LMU Munich, Munich, Germany; 30 Department of Psychology, University of Regensburg, Regensburg, Germany; 31 Department of Psychosomatic Medicine and Psychotherapy, University of Tuebingen, Tübingen, Germany; 32 German Centre for Mental Health (DZPG), Tübingen, Germany; 33 Departments of Psychiatry and Epidemiology, Columbia University Irving Medical Center, New York, NY USA; 34 Department of Child and Adolescent Psychiatry, Medical University Vienna, Vienna, Austria; 35 Department of Psychiatry and Behavioral Science, University of North Dakota, Fargo, ND USA; 36 Centre for Health Performance and Wellbeing, Anglia Ruskin University, Cambridge, UK; 37 Department of Public Health, Faculty of Medicine, Biruni University, Istanbul, Turkey; 38 Regional Centre for the Treatment of Eating Disorders and On Track Program, The Ottawa Hospital, Ottawa, ON Canada; 39 Department of Child and Adolescent Psychiatry, Charité Universitätsmedizin, Berlin, Germany

**Keywords:** Eating disorders, Duration of untreated eating disorders, Treatment delay, Early intervention, Meta-analysis

## Abstract

**Introduction:**

Eating disorders (EDs) substantially impair physical health, psychological well-being, and social functioning. Shorter untreated illness duration has been proposed to be associated with better outcomes, though evidence for this relationship remains mixed. Nonetheless, delays in accessing treatment remain common and contribute to prolonged suffering and risk of lasting harm. This study aimed to estimate the duration of untreated eating disorder (DUED) and examine potential moderators.

**Methods:**

A PRISMA 2020-compliant systematic review and meta-analysis was conducted. Searches were performed from inception to March 2026 in PubMed, Embase, PsycINFO, and CINAHL. Random-effects models were used to estimate pooled DUED of available studies, with subgroup and meta-regression analyses to explore moderators. Study quality and risk of bias were assessed using National Institutes of Health (NIH) tools.

**Results:**

Of 2,776 records identified, 29 studies (*n* = 17,433) were included. The pooled DUED of available studies was 21.9 months (95% CI 18.0–25.8), with substantial heterogeneity (I² = 99.7%). In subgroup analyses, DUED was longer in bulimia nervosa than anorexia nervosa (41.5 vs. 19.9 months). Meta-regression identified mean age as a significant moderator (β = 1.91, 95% CI 1.1 to 2.7, *p* < 0.001), indicating longer delays in older individuals. DUED also differed across country, continent, age group, and study design, while decade subgroup estimates were lower in studies from the 2020s than the 2010s.

**Conclusion:**

Individuals with EDs experience substantial delays before accessing treatment, with untreated illness exceeding 20 months on average. Older age was associated with longer DUED, suggesting potential gaps in detection and help-seeking among adults. DUED varies across demographic, geographic and methodological factors. While decreasing DUED over time is encouraging, these findings highlight the need for earlier identification and streamlined care pathways to reduce delays and improve outcomes in people with ED.

**Supplementary Information:**

The online version contains supplementary material available at https://doi.org/10.1186/s40337-026-01731-6.

## Abstract (plain language summary)

Eating disorders are serious mental health conditions that can have lasting effects on health and wellbeing. One factor known to affect recovery is how long a person goes without receiving treatment after their illness begins, referred to as the duration of untreated eating disorder (DUED). Shorter treatment delays are generally associated with better outcomes, yet many individuals wait months or years before accessing care. This study combined data from 29 studies involving over 17,000 individuals to estimate how long people typically wait before accessing care. On average, individuals waited approximately 22 months, with notably longer delays in bulimia nervosa (42 months) than anorexia nervosa (20 months). Older individuals tended to experience longer delays, and studies from the 2020s reported shorter delays than those from the 2010s, possibly reflecting improvements in awareness and early intervention programmes. Most included studies came from European countries, limiting the generalisability of findings. These results highlight the need for continued investment in early identification and accessible care pathways for eating disorders.

**Supplementary Information:**

The online version contains supplementary material available at https://doi.org/10.1186/s40337-026-01731-6.

## Introduction

Eating disorders (EDs) are severe psychiatric conditions associated with substantial morbidity and markedly elevated premature mortality compared with the general population [1]. Although a range of psychological and multidisciplinary treatments are recommended [2], treatment effectiveness varies across diagnoses. Pharmacological and non-pharmacological interventions have demonstrated benefit particularly for bulimia nervosa (BN) and binge eating disorder (BED) [3]. In contrast, for anorexia nervosa (AN), no pharmacological treatment has shown consistent efficacy, and no single outpatient psychological intervention has reliably outperformed treatment as usual in adults [3, 4]. These challenges highlight the complexity of EDs and the importance of timely, evidence-based, multidisciplinary care.

Early intervention is widely recognized as a key determinant of improved outcomes in EDs. However, delays in accessing specialized treatment remain common and are influenced by multiple factors [5]. Delays in treatment initiation may allow symptoms to become more entrenched, increase medical and psychiatric complications, reduce responsiveness to intervention and increase long-term healthcare costs [6]. Although the relationship between illness duration and treatment outcomes remains mixed across studies [7–9], reducing delays in access to care may help minimize suffering and prevent the progression of more severe or enduring illness. Understanding the duration of untreated eating disorders (DUED) and the factors associated with treatment delays therefore remains clinically and public health relevant.

Evidence from structured early-intervention programmes underscores the clinical importance of reducing DUED. The First Episode Rapid Early Intervention for Eating Disorders (FREED) model, in the UK, has been shown to shorten DUED, improve clinical outcomes, and reduce reliance on costly inpatient care [10, 11]. Similar rapid-access pathways are being developed internationally [12, 13]. Systematic evidence on the magnitude and moderators of DUED is therefore needed to inform the design and targeting of such programmes.

The concept of DUED captures this interval and may represent a clinically meaningful and potentially modifiable target for improving outcomes [14]. If shorter DUED is associated with better prognosis independent of treatment type, understanding its magnitude and variability across populations is essential for informing early intervention strategies and health service planning. Reducing DUED may represent a key and potentially modifiable target for early intervention strategies and health system planning aimed at improving outcomes in individuals with EDs. In particular, the extent to which DUED varies across diagnoses, populations, and study characteristics, and the factors contributing to this variability, remain unclear. Importantly, the evidence base for universal eating-disorder screening remains limited; the United States Preventive Services Task Force concluded that evidence is insufficient to determine the balance of benefits and harms of screening asymptomatic adolescents and adults, highlighting the need to identify targeted pathways that demonstrably shorten DUED [15].

To date, the most recent systematic review on DUED searched the literature up to December 2019 and identified 14 studies (reported in 17 publications) [14]. That review focused primarily on AN and did not include meta-regression analyses to explore potential sources of heterogeneity. Since then, additional studies have been published [15–18], providing an opportunity to update and expand the evidence base. Therefore, the present systematic review and meta-analysis aim to estimate the DUED across EDs, and examine potential moderators of DUED using meta-regression and subgroup analyses.

## Methods

This systematic review was conducted using the Preferred Reporting Items for Systematic Reviews and Meta-Analyses (PRISMA) guidelines [19]. Searches were conducted from inception to March 2026 from the following databases: PubMed, Embase, PsycINFO, and CINAHL using search terms synonymous with EDs and duration of untreated illness, with full, database specific search strategies being available in the Supplementary Materials. In addition to the electronic database searches, backward citation searching of the reference lists of all included studies was performed to identify additional potentially eligible studies. Titles and abstracts, followed by full text screening, were conducted by three independent authors (MT, AMM, GDG), with disagreements resolved by a senior author (MS) when necessary.

### Eligibility criteria

Studies were selected according to pre-specified eligibility criteria, using the PICO framework [20]. The eligible population comprised individuals of any age, sex, and clinical setting diagnosed with or meeting criteria for any eating disorder, including AN, BN, BED, avoidant/restrictive food intake disorder (ARFID), other specified feeding or eating disorder (OSFED) or eating disorder not otherwise specified (EDNOS). Diagnoses based on any established criteria (e.g., DSM, ICD) were eligible. The primary exposure of interest was DUED, defined as the time elapsed between illness onset and first treatment contact or initiation of care. In first-episode samples, DUED was operationalised as the duration of illness to date. No comparator group was required for inclusion. The primary outcome was mean (or median) DUED in months; secondary outcomes included subgroup DUED estimates by diagnosis, age, sex, and geographic region. Where available, differences in DUED across these subgroups, as well as by geographic region and time period, were examined as potential moderators in subgroup and meta-regression analyses.

DUED was extracted according to the definition provided in each included study. When studies of first-episode eating disorders reported duration of illness rather than an explicit DUED measure, duration of illness was accepted as the closest available proxy for DUED, as these studies generally enrolled individuals during their first episode of illness. However, because prior healthcare contacts or treatment before study enrolment were not consistently reported, this operationalisation may not be fully equivalent to DUED across all studies and may have contributed to methodological heterogeneity. All observational (cross-sectional, cohort, case-control) and interventional study designs were eligible. No language restrictions were applied; publications in all languages were eligible for inclusion. Following the screening process, data were extracted independently by two authors (GDG, MT) using a pre-specified template. Extracted variables included the median year and country of data collection, type of ED, DUED, age, gender, sample size, body mass index (BMI), and other psychopathological features.

### Statistical analysis

All statistical analyses were conducted using R [21]. Mean DUED and standard deviation (SD) were pooled using a random effects meta-analysis using the Restricted Maximum Likelihood method to weight studies. If data for DUED were reported as medians, these were converted into means and SDs using the Wan et al. formulas [22]. If more than one definition of DUED were present in any individual study, to prevent multiple populations from the same study being included in pooled analyses, only the definition of symptom onset to treatment was used. Heterogeneity was determined using the I^2^ statistic, with < 50% being classified as low, 51–75 moderate, and > 75% being classified as high heterogeneity [23]. Publication bias was determined by the Egger’s test [24] if k studies ≥ 10, and via funnel plot if k studies ≤ 10 [25]. If significant publication bias was found, we conducted a trim-and-fill analysis [26] to estimate DUED in the absence of publication bias.

To investigate potential sources of heterogeneity, univariate meta-regression analyses were conducted for prespecified moderators with sufficient data (≥ 10 studies), including mean age, mean BMI, percentage of female participants, year of publication, and sample size. Furthermore, prespecified subgroup analyses were conducted for categorical variables, including diagnosis, country, continent, decade of data collection, age group, and study design, to generate stratified pooled estimates and explore potential sources of between-study heterogeneity [27].

### Quality assessment and risk of bias

Risk of bias and methodological quality of included studies were assessed using the National Institutes of Health (NIH) Quality Assessment Tools for observational cohort and cross-sectional studies [28]. Each study was evaluated across predefined domains according to NIH guidance. The tool comprises 14 criteria rated Yes/No/Not Applicable (range 0–14 points). Studies scoring 11–14 were classified as Good, 5–10 as Fair, and 0–4 as Poor. Assessments were conducted independently by two reviewers (GDG, MT), with disagreements resolved through discussion and, when necessary, consultation with a third reviewer (MS).

Between-study heterogeneity was examined using the I² statistic and visual inspection of confidence interval overlap in forest plots. Sensitivity analyses were conducted to explore the robustness of pooled estimates. Potential publication bias was assessed using funnel plot inspection and Egger’s regression test where sufficient studies were available.

## Results

**Fig. 1:**
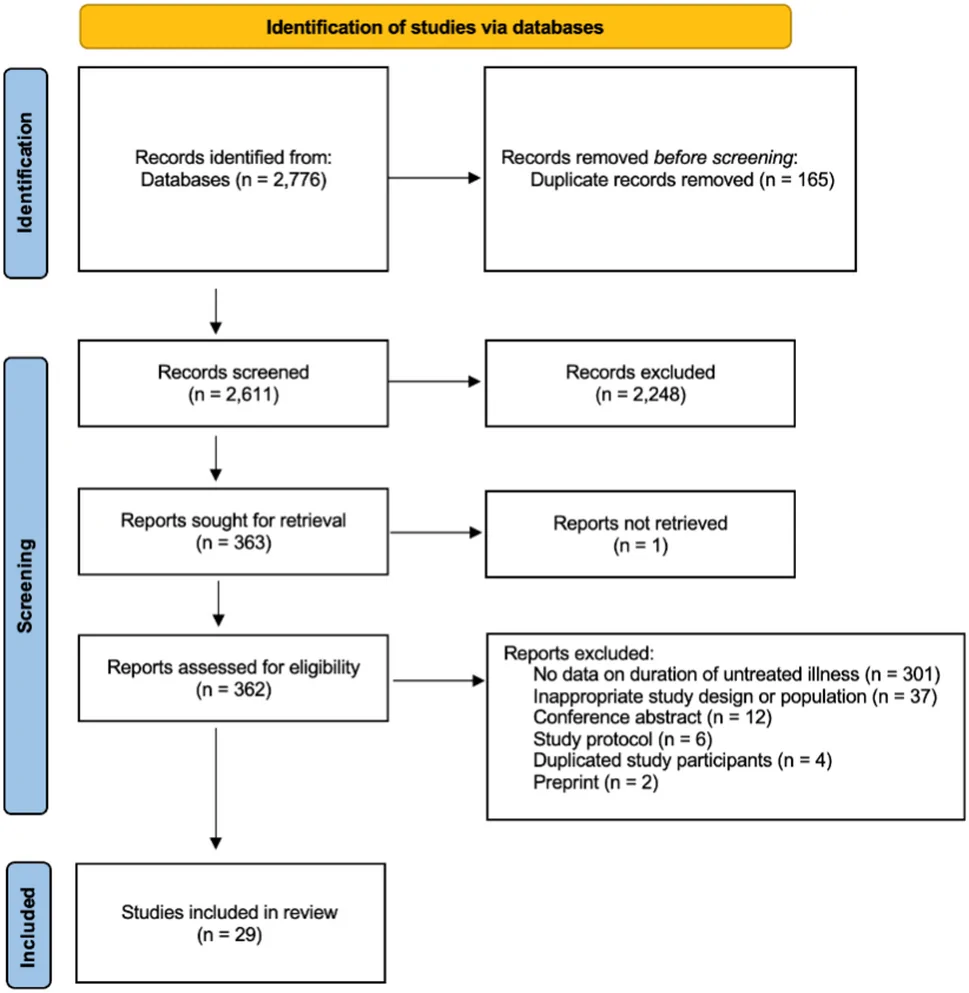
PRISMA flowchart of included studies

After 2776 initial search results, 165 were automatically removed as duplicates, leaving 2611 title and abstracts to be evaluated. Following initial screening, 362 full texts were reviewed, with 29 studies being included following full-text assessment. Reasons for full text exclusion can be found in Fig. 1, and the complete list of excluded studies can be found in Supplementary Table 1. The meta-analysed studies had a total of 17,433 participants, with a mean age of 18.6 (SD = 4.4; median age per study = 19.2), and a mean of 92% (SD = 19%; median 95% per study) were female. Most of the included studies were conducted in Europe (72%), followed by Asia (14%), South America (10%), and North America (3%) with data being collected from 1994 to 2024. Twenty-three studies were cross-sectional in design (79%), with two respective studies incorporating case-control and qualitative study designs (7% each) and one randomized control trial and one non-randomized intervention study designs (3% each). Regarding types of ED included in samples, 90% included patients with AN, 38% with OSFED/EDNOS, 31% included patients with BN, 20% included BED, and 14% included ARFID. Regarding definitions of DUED, 79% of studies did not define DUED, see Table 1 for full demographic information. For information on inclusion and exclusion criteria of the individual studies included in this review, see Supplementary Table 2.

**Table 1:** Descriptive characteristics of studies included in the systematic review and meta-analyses

Authors (publication year)	Year of data collection (median)	Country	Study design	ED diagnostic criteria	ED types included in sample	DUED definition	*N*	Mean age (SD)	Mean BMI (SD)	Percentage female	Quality rating
Brown et al. (2018) [10]	2014	UK	Cross-sectional	DSM-5	AN, BN, BED, OSFED	Length of time (in months) between ED onset and start of evidence-based treatment	Audit = 89 FREED = 51	Audit = 20.4 (1.9) FREED = 20.6 (2.5)	Audit = 19.7 (4.3) FREED = 20.2 (4.3)	Audit = 97.7% FREED = 96%	Fair
Buhren et al. (2013) [29]	2009	Germany	Cross-sectional	DSM-IV	AN	Time between the beginning of weight loss and admission to hospital	116*	15.2 (1.7)	14.8 (1.2)	100%	Fair
Caldiroli et al. (2024) [30]	2023	Italy	Cross-sectional	DSM-5	AN; BN; OSFED; BED	Unspecified symptom onset to unspecified endpoint	51	24.8 (8.2)	22.9 (6.8)	94%	Fair
Campbell et al. (2022) [31]	2020	Ireland	Cross-sectional	Clinical diagnosis (e.g. medical records)	AN; ARFID; OSFED/EDNOS	Duration of weight loss (months) to pre-referral (months)	128	14.5 (2.0)	NR	92.0%	Fair
Castañeda et al. (2024) [32]	NR	Chile	Qualitative	DSM-5	AN; BN; OSFED; BED	Time between the onset of symptoms and the initiation of appropriate treatment.	22	21.8 (7.5)	19.7 (5.1)	96%	Good
Chew et al. (2020) [33]	2014	Singapore	Cross-sectional	DSM-5	AN; ARFID; OSFED/EDNOS	Unspecified symptom onset to unspecified endpoint	274	13.9 (15)	NR	91%	Good
De Stefani et al. (2023) [34]	NR	Brazil	Cross-sectional	DSM-5	AN; BN	Unspecified symptom onset to unspecified endpoint	271	21.5 (9.0)	18.7 (5.3)	90%	Fair
Del Berrio et al. (2022) [35]	2018	Spain	Case-control	Clinical diagnosis (e.g. medical records)	AN; BN; OSFED/EDNOS; BED	Unspecified symptom onset to a contact from a specialist service	97	AN = 18.4 (3.1) BN = 19.3 (3.6) OSFED = 19.1 (3.4) BED = 20.0 (2.3)	AN = 17.1 (1.3) BN = 23.7 (4.6) OSFED = 22.4 (2.6) BED = 30.5 (8.1)	NR	Fair
Flynn et al. (2020) [36]	2013 and 2017	UK	Cross-sectional	DSM-5	AN, BN, BED, OSFED	Length of time (in months) between ED onset and start of evidence-based treatment	FREED = 278 TAU = 224	FREED = 20.1 (2.39) TAU = 20.2 (2.43)	NR	FREED = 93.16% TAU = 96.42	Good
Gallagher et al. (2025) [18]	2022	UK	Cross-sectional	DSM-5	AN, BN, BED, OSFED	Length of time between the onset of clinically significant ED symptoms and the initiation of evidence-based treatment.	Pre Covid-19 = 141 During Covid-19 = 2061 After Covid-19 = 9237	Pre Covid-19 = 19.6 (2.5) During Covid-19 = 18.3 (2.1) After Covid-19 = 19.2 (2.3)	NR	NR	Fair
Guerrero et al. (2022) [37]	NR	Spain	Case-control	DSM-5	AN; BN; OSFED	Time between the onset of illness and the initiation of treatment	48	NR	NR	79%	Fair
Gumz et al. (2018) [38]	Pre-intervention: 2013 Post-intervention: 2014	Germany	Non-randomised intervention	DSM-5	AN	Symptom onset to treatment; Symptom onset to first contact with healthcare (both types analysed separately)	77	Pre-intervention: 21.5 (7.2) Post-intervention: 17.2 (1.7)	Pre-intervention: 17.2 (1.7) Post-intervention: 16.9 (1.3)	100%	Fair
Gumz et al. (2023) [39]	2021	Germany	Cross-sectional	DSM-5	AN	The interval between the onset of ED symptoms and start of specialized treatments	125	19.2 (4.1)	15.7 (1.9)	100%	Good
Harada et al.(2020) [40]	Group 1: 1994 Group 2: 2004 Group 3: 2014	Japan	Cross-sectional	DSM-5	AN-R	Unspecified symptom onset to the first medical treatment	996	Group 1: 20.3 (5.1) Group 2: 22.5 (7.8) Group 3: 25.1 (10.6)	NR	100%	Good
Herpertz-Dahlmann et al. (2014) [41]	2009	Germany	Randomized control trial	DSM-IV	AN	Unspecified symptom onset to unspecified endpoint	172	15.3 (1.5)	15.0 (1.4)	100%	Good
Jaite et al. (2019) [42]	2017	Germany	Retrospective cohort	NR	AN	Weight loss (not otherwise stated)	Children = 72; adolescents = 217	Children = 12.4 (0.9); adolescents = 15.0 (1.1)	Children = 14.3 (1.3); adolescents = 15.5 (1.2)	Children = 97.2%; adolescents = 97.7%	Good
Kastner et al. (2021) [43]	NR	Germany	Qualitative	Clinical diagnosis (e.g. medical records)	NR	Unspecified symptom onset to unspecified endpoint	10	Adults 23.3 (7.5); Adolescents 15.8 (1.5)	Adults 15.9 (2.3); Adolescents 15.6 (1.4)	NR	Fair
Kwok et al. (2019) [44]	2003	Singapore	Cross-sectional	DSM-IV-TR	AN	Unspecified symptom onset to unspecified endpoint	438	Early onset 11.5 (1.1) Late onset 15.2 (1.6)	Early onset 14.4 (2.0) Late onset 14.9 (1.8)	Early onset 91.7% Late onset 95.7%	Fair
Lieberman et al. (2019) [45]	2013	Canada	Cross-sectional	DSM (unspecified version)	AN; ARFID	Parent report of symptom onset (e.g., food avoidance and food restriction) to unspecified endpoint	106	11.3 (0.9)	AN 15.8 (2.1); ARFID 14.6 (1.6)	AN 85.7%; ARFID 75.9%	Good
McClelland et al. (2020) [46]	2011	UK	Cross-sectional	Questionnaire with cut-off, not based on DSM or ICD criteria	AN; BN; OSFED/EDNOS	Unspecified symptom onset to unspecified endpoint	322	No contact with AMHS: 15 (1.5) Low, medium, and high use of AEDS: 16 (1.5) Local non-ED AMHS: 16 (1.5) Non-local unspecified AMHS: 16.3 (0.7)	NR	NR	Fair
Monteleone et al. (2023) [5]	2020	Multiple European countries	Cross-sectional	DSM-5	AN; BN; BED; OSFED/EDNOS	Months between the onset of ED and access to ED unit	409	26.6 (11.2)	NR	93.6%	Good
Neubauer et al. (2014) [47]	NR	Germany	Cross-sectional	DSM-IV and ICD-10	AN	Time between onset and presentation to first ED-specific treatment	140	NR	NR	100%	Fair
Ng et al. (2018) [48]	2011	Singapore	Cross-sectional	DSM-5	AN; BN; OSFED/EDNOS	Unspecified symptom onset to unspecified endpoint	257	20.5 (7.1)	17.8 (4.2)	92.6%	Fair
Reuter et al. (2021) [49]	2019	Germany	Cross-sectional	Clinical diagnosis (e.g. medical records)	AN	Unspecified symptom onset to unspecified endpoint	75	21.4 (7.4)	NR	100%	Good
Rosello et al. (2021) [16]	2013	UK	Cross-sectional	ICD-10	AN; BN	Parent report of symptom onset such as: Concerns about weight; Concerns about shape; Changes in eating patterns (the three types were analysed separately)	78	14.9 (1.6)	NR	94.0%	Good
Salas et al. (2011) [50]	2005	Chile	Cross-sectional	DSM-IV	AN	Unspecified symptom onset to unspecified endpoint	38	14.4 (2.2)	NR	0%	Fair
Schlegl et al. (2019) [51]	2011	Germany	Cross-sectional	ICD-10	AN; BN	Symptom onset to diagnosis; Symptom onset to treatment; Diagnosis to treatment (all three analysed separately)	206	AN: 24.3 (7.7) BN: 25.4 (8.3)	AN: 16.3 (1.9) BN: 22.1 (3.8)	AN: 96.4% BN: 98.5%	Good
Valenta et al. (2025) [52]	2024	Italy	Cross-sectional	DSM-5	AN; BN; OSFED; BED	Symptom onset to first contact with healthcare professional and symptom onset to first treatment	174	23.5 (5.4)	20.0 (5.9)	94%	Good
Weigel et al. (2014) [53]	2012	Germany	Cross-sectional	DSM-IV	AN	Length of time between the onset of an illness and the initiation of recommended treatment	58	22.3 (7.8)	16.3 (2.0)	100%	Fair

*AEDS* adolescent eating disorder service, *AMHS* adult mental health service, *AN* anorexia nervosa, *AN-R* anorexia nervosa resistant, *ARFID* avoidant/restrictive food intake disorder; *BED* binge eating disorder, *BN* bulimia nervosa, *DSM* diagnostic and statistical manual, *DUED* duration of untreated eating disorder, *ED* eating disorder, *EDNOS* eating disorder not otherwise specified, *FREED* First Episode Rapid Early Intervention in Eating Disorders, *ICD* international classification of diseases, *NR* not reported, *OSFED* other specified feeding or eating disorder, *SD* standard deviation, *TAU* treatment as usual

*Only the local Aachen subsample was included (*n* = 116 of 158 total); 42 patients overlapping with the multi-site RCT sample (Herpertz-Dahlmann et al., 2014) were excluded to preserve sample independence

### Meta-analysis results

**Fig. 2:**
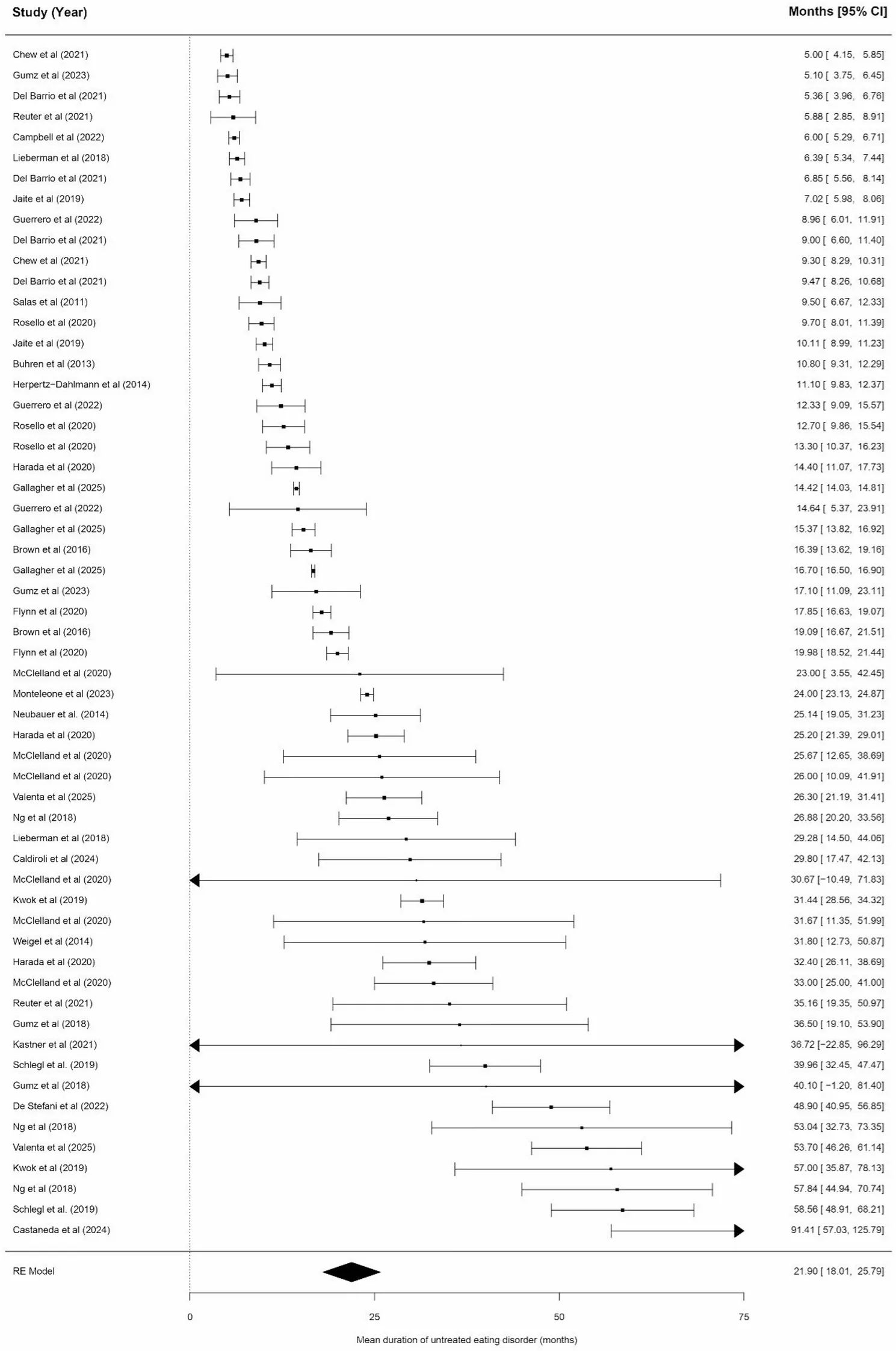
Forest plot showing duration of untreated illness across all included studies

Pooling data across all included studies, the random-effects meta-analysis yielded a mean DUED of 21.9 months (k = 29, 95% CI 18.0 to 25.8; Fig. 2). Heterogeneity was substantial (I² = 99.7%). The Egger test indicated publication bias (Egger’s *p* < 0.001, see Supplementary Fig. 1). Trim-and-fill analyses did not materially alter results.

### Subgroup meta-analysis results

Subgroup analyses (Table 2) indicated significant differences in DUED across country, continent, decade, age group, study design, and diagnosis (all QM *p* < 0.001). However, the proportion of heterogeneity explained by these factors was modest (R² range: 1.7% to 34.7%). At the country level, pooled estimates ranged from 6.0 months in Ireland to 48.9 months in Brazil, although several categories were informed by only one study. Across continents, pooled DUED for available studies was 19.1 months in Europe, 29.5 months in Asia, and 47.4 months in South America. DUED was higher in studies from the 2010s than the 2020s, in adults than adolescents, and varied across study design and diagnosis. By diagnosis, pooled DUED was 19.9 months for AN, 41.5 months for BN, 20.9 months for mixed samples, and 28.1 months for OSFED/EDNOS, while ARFID and BED estimates were based on single studies.

**Table 2:** Subgroup meta-analysis

Variable	Subgroup	k outcome (*n* studies)	Mean DUED	CI	Test for differences (QM)	*R* ^2^
Country	Brazil	1 (1)	48.9	40.9 to 56.8	205.78 *p* < 0.001	34.7%
	Canada	2 (1)	16.6	− 5.7 to 38.9		
	Chile	2 (2)	48.6	− 31.6 to 128.7		
	China	2 (1)	7.1	2.9 to 11.3		
	Germany	15 (10)	22.4	13.6 to 31.1		
	Ireland	1 (1)	6.0	5.2 to 6.7		
	Italy	4 (3)	33.2	19.6 to 46.9		
	Japan	3 (1)	23.7	13.5 to 33.9		
	Singapore	5 (2)	43.2	29.4 to 56.9		
	Spain	7 (2)	8.6	6.7 to 10.4		
	UK	16 (5)	17.3	14.72 to 19.92		
Continent	Asia	10 (4)	29.5	18.2 to 40.9	132.09 *p* < 0 0.001	3.0%
	Europe	43 (21)	19.1	15.3 to 22.9		
	North America	2 (1)	16.6	− 5.7 to 38.9		
	South America	3 (3)	47.4	2.5 to 92.3		
Decade	2010	20 (12)	27.1	19.4 to 34.9	128.61 *p* < 0.001	3.6%
	2020	38 (17)	19.0	14.9 to 23.2		
Age group	Adolescent	12 (10)	11.1	7.3 to 15.0	145.71 *p* < 0.001	12.3%
	Adults	7 (5)	27.6	17.6 to 37.7		
	Children	11 (5)	24.2	18.8 to 29.6		
	Mixed	28 (14)	25.7	19.3 to 32.0		
Study design	Case control	7 (2)	8.6	6.7 to 10.4	157.91 *p* < 0.001	16.6%
	Cross-sectional	46 (23)	23.1	18.8 to 27.3		
	Non-randomized intervention	2 (1)	37.0	21.0 to 53.0		
	Randomized control trial	1 (1)	11.1	9.8 to 12.3		
	Qualitative	2 (2)	69.7	17.2 to 122.1		
Diagnosis	AN	23 (16)	19.9	14.5 to 25.3	129.58 *p* < 0.001	1.7%
	ARFID	1 (1)	29.2	14.5 to 44.1		
	BED	1 (1)	9.0	6.6 to 11.4		
	BN	3 (3)	41.5	9.2 to 73.7		
	Mixed ED’s	28 (14)	20.9	16.0 to 25.8		
	OSFED or EDNOS	2 (2)	28.1	− 18.6 to 74.7		

*AN* anorexia nervosa, *ARFID* avoidant/restrictive food intake disorder, *BED* binge eating disorder, *BMI* body mass index, *BN* bulimia nervosa, *CI* confidence interval, *DUED* duration of untreated eating disorder, *EDNOS* eating disorder not otherwise specified, *OSFED* other specified feeding or eating disorder

### Meta regression results

Meta-regression analyses in the total sample identified mean age as a significant moderator of DUED (β = 1.91, 95% CI 1.1 to 2.7, *p* < 0.001; Table 3 and Supplementary Fig. 2). In contrast, mean BMI, sex (percent female), percent inpatient, publication year, and sample size were not significantly associated with DUED.

**Table 3:** Meta regression results

	Moderating variable	*k* outcomes (*n* studies)	β co-efficient (95% CI)	p-value	I^2^
Total sample	Mean age	57 (28)	1.91 (1.1 to 2.7)	< 0.001	99.6
	Mean BMI	37 (19)	0.42 (− 1.2 to 2.0)	0.607	99.5
	Sex (percent female)	44 (25)	0.1 (− 0.1 to 0.3)	0.407	99.6
	Percent inpatient	24 (13)	− 0.21 (− 0.5 to 0.1)	0.162	99.7
	Publication year	58 (29)	0.17 (− 1.1 to 1.4)	0.79	99.7
	Sample size	58 (29)	0 (0 to 0)	0.738	99.6
AN subgroup	Mean age	22 (15)	1.64 (0.5 to 2.7)	0.004	98.7
	Mean BMI	21 (14)	− 1.21 (− 6.8 to 4.4)	0.672	99.3
	Sex (percent female)	21 (14)	− 0.23 (− 1.8 to 1.3)	0.773	99.1
	Percent inpatient	17 (11)	− 0.14 (− 0.4 to 0.1)	0.251	99.3
	Publication year	23 (16)	− 0.58 (− 2.5 to 1.4)	0.565	99.2
	Sample size	23 (16)	0.03 (0 to 0.1)	0.136	99
Mixed ED’s subgroup	Mean age	28 (14)	1.88 (0.8 to 2.9)	< 0.001	99.7
	Sex (percent female)	19 (12)	0.1 (− 0.1 to 0.3)	0.408	99.7
	Publication year	28 (14)	1.24 (− 0.2 to 2.7)	0.101	99.8
	Sample size	28 (14)	0 (0 to 0)	0.687	99.7

*AN* anorexia nervosa, *BMI* body mass index, *ED* eating disorder

The DUED of people with AN was 19.9 months (k = 16, 95% CI 14.5 to 25.4 months), see Supplementary Fig. 3, with high heterogeneity (I^2^ = 99.2%) and evidence of publication bias (Egger’s *p* < 0.001, see Supplementary Fig. 4). Within the AN subgroup, meta-regression analyses showed that mean age was significantly associated with DUED (β = 1.64, 95% CI 0.5 to 2.7, *p* = 0.004; see Table 3 and Supplementary Fig. 5), whereas mean BMI, sex (percent female), percent inpatient, publication year, and sample size were not significant moderators.

The DUED in the mixed-diagnosis subgroup was 20.9 months (k = 14, 95% CI 16.0 to 25.8 months), see Supplementary Fig. 6, with high heterogeneity (I^2^ = 99.8%) and evidence of publication bias (Egger’s *p* < 0.001, see Supplementary Fig. 7). In the mixed-diagnosis subgroup, meta-regression analyses were conducted for moderators with data available from at least 10 studies. Mean age was significantly associated with DUED (β = 1.88, 95% CI 0.8 to 2.9, *p* < 0.001, see Table 3 and Supplementary Fig. 8), whereas sex, publication year, and sample size were not significant moderators. Meta-regression was not conducted for mean BMI or percent inpatient due to insufficient data.

### Study quality assessment

The methodological quality of the included studies was assessed using the NIH Quality Assessment Tool for Observational Cohort and Cross-Sectional Studies [54]. Of the 29 included studies, 13 studies were rated as good quality and 16 as fair quality, with no studies rated as poor quality (Table 1). The most common methodological limitations included incomplete reporting of participation rates, lack of sample size justification, and limited adjustment for potential confounding variables. Overall, the included evidence was considered to be of moderate-to-high methodological quality.

### Sensitivity analyses

Sensitivity analyses were performed using a leave-one-out approach, whereby each study was sequentially excluded and the pooled DUED estimate recalculated to assess the influence of individual studies on the overall pooled estimate and between-study heterogeneity, demonstrated that exclusion of any individual study did not materially alter the pooled estimate or the magnitude of between-study heterogeneity (Supplementary Tables 3–5). This included the Gallagher et al. [18] study, which contributed the largest proportion of participants (*n* = 11,439), representing more than half of the total sample included in the meta-analysis, but had minimal influence on the overall pooled estimate. Influence diagnostics and residual plots identified a small number of potentially influential studies (Supplementary Fig. 9); however, exclusion of these studies did not meaningfully change the results, supporting the robustness of the findings.

## Discussion

This systematic review and meta-analysis provide a comprehensive and up-to-date synthesis of the DUED. Across included studies, the pooled DUED was approximately 21.9 months, indicating that individuals with EDs experience substantial delays before accessing treatment. In subgroup analyses the pooled DUED was 19.9 months for AN and 41.5 months for BN, suggesting that prolonged delays are also present within this diagnostically specific group. However, given the high heterogeneity observed across studies (I² = 99.7%), the pooled estimate should be interpreted with caution. This level of heterogeneity suggests that rather than representing a precise clinical benchmark, the pooled estimate provides an indication of the average magnitude of treatment delays for available studies, while the subgroup and meta-regression analyses offer more informative insights into the clinical, geographical, and methodological factors contributing to this variability.

A major source of heterogeneity was the lack of a standardized operational definition of DUED. Approximately 79% of included studies did not explicitly define DUED, and substantial variation existed in how both illness onset and treatment initiation were operationalised. Although most studies defined illness onset according to the emergence of eating disorder symptoms [29, 33, 35], others used more specific indicators, such as weight loss [42], concerns about weight or shape [16], or parent- or patient-reported symptom onset [44, 45]. Similarly, treatment endpoints ranged from first healthcare contact to treatment initiation, while many studies did not clearly specify the endpoint used. Consequently, studies may have captured different stages of the care pathway, limiting comparability across settings. Greater consistency in defining and reporting DUED is therefore essential to improve future evidence synthesis.

Recognizing that DUED in EDs is considerably longer than the duration of untreated psychosis, typically reported as 40–44 weeks (9–10 months) [55], underscores the challenge of early detection in this field. Across the available literature, several reasons may contribute to these delays in treatment. Some studies described minimisation of symptoms and symptom secrecy as central motives for postponing help-seeking [39, 51]. Individuals with AN and BN may perceive symptoms as manageable or under personal control, delaying disclosure to family members or healthcare professionals [35]. In some cases, restrictive eating behaviours may also be reinforced by a temporary sense of achievement or control [39]. DUED appeared longer in BN compared to AN, which may reflect differences in illness recognition and help-seeking pathways [51]. Individuals with BN often present with fewer visible physical manifestations of illness, such as low BMI or overt signs of malnutrition [56], potentially delaying identification and referral to specialized services. This difference may additionally reflect diagnostic uncertainty or definitional issues. Given the relapsing nature of BN, studies measuring DUED from age at first onset rather than from the current untreated episode may systematically report longer delays [34]. Future research should therefore clearly distinguish between lifetime and current untreated duration.

Individual and social factors also appear to contribute substantially to prolonged DUED. Qualitative studies highlighted stigma, shame, and fear of judgment as pervasive barriers to treatment-seeking [43, 52]. These barriers may be particularly evidenced among males and adults, who are less likely to identify themselves with stereotypical ED presentations and remain underrepresented in both research and clinical settings [16, 50]. Adolescents may benefit from earlier detection through parental or school involvement, whereas adults often rely more heavily on self-recognition and may delay seeking care due to ambivalence, stigma, or competing personal responsibilities [49]. Recent studies have similarly identified stigma, shame, and guilt as major patient-reported barriers, alongside limited ED knowledge among healthcare professionals as an important clinician- and caregiver-reported barrier [57, 58].

Certain clinical characteristics may also delay the recognition of an ED. Although BMI was not a significant moderator in the present meta-regression, individual studies [39] suggest that higher BMI or absence of overt medical complications may reduce clinical recognition and delay referral. Similarly, Lieberman et al. [45] observed that children with ARFID were diagnosed later than those with AN, highlighting the limited awareness and recognition of non-AN ED presentations.

Structural and system-level factors were repeatedly identified as critical barriers. Long waiting lists, scarcity of specialized services, and fragmented referral pathways were major contributors to delay [5, 52]. Castañeda et al. [32] described how limited clinician training, economic barriers, and regional disparities hindered timely access to care. Similarly, Gómez del Barrio et al. [35] reported that most young patients had multiple health-service contacts in the year prior to diagnosis without receiving appropriate referrals. These findings suggest that a substantial portion of DUED occurs before the first specialist assessment, as also noted in Gumz et al. [39] and Neubauer et al. [47]. EDs typically require a multidisciplinary approach, involving physicians, psychiatrists, psychologists, and nutrition specialists [59]. Individuals with EDs often utilize both mental and physical healthcare services well before diagnosis [60]. A study from Taiwan found that nearly all patients later diagnosed with AN or BN sought care for somatic complaints in the year prior [61]. Initial diagnoses are rarely specific to EDs, instead often including anxiety, depressive, personality, or neurotic disorders [62]. This suggests that alternative diagnoses and the presence of affective symptoms [5] can delay accurate identification and timely specialist referral.

Meta-regression analyses further indicated that mean age was positively associated with DUED, suggesting that older individuals tend to experience longer delays before accessing treatment, a finding consistent with previous studies [44, 47, 63]. Adolescents often benefit from external detection by parents, teachers, or pediatric providers, which facilitates earlier referral [16, 64]. In contrast, adults face more complex barriers to help-seeking, as they may lack supportive observers, internalize stigma, and face competing personal and professional responsibilities that delay treatment initiation [52]. Differences in healthcare organisation may also contribute, as services for adolescents are often more structured than those available for adults [65].

This association may also reflect differences in diagnostic composition. Bulimic-spectrum disorders are often diagnosed later than anorexia nervosa and are generally associated with longer DUED [34]. Individuals with BN often present without obvious physical signs of illness and may initially receive alternative psychiatric or medical diagnoses, delaying recognition and referral [35]. Consequently, studies including a greater proportion of individuals with BN also tended to include older participants and longer DUED estimates, making it difficult to disentangle the independent effects of age and diagnosis [34].

However, the observed age effect should be interpreted cautiously. Because the present meta-regression was based on aggregate study-level data, residual confounding cannot be excluded. An alternative explanation is a historical cohort effect, whereby older participants developed their eating disorder during periods when awareness was lower and access to specialist services was more limited [10, 36]. This interpretation is supported by our subgroup analyses, which showed shorter DUED estimates in studies conducted during the 2020s [18, 34, 37, 40] than those from the 2010s [10, 29, 38, 47]. The establishment and expansion of structured early intervention pathways, such as the FREED model in the United Kingdom [10], introduced in 2015 and subsequently scaled nationally, may have contributed to this temporal shift, although this interpretation remains speculative in the absence of service-level data from individual studies. In addition, retrospective ascertainment of illness onset is susceptible to telescoping bias [63], and DUED is mathematically constrained by age at assessment, potentially exaggerating the observed association. Future individual participant data meta-analyses or prospective studies are needed to distinguish age effects from cohort effects and better characterise the relationship between age and treatment delay.

Cultural context also shapes DUED. Studies from Asia and Latin America demonstrate that sociocultural norms and healthcare disparities may influence recognition and referral. Chew et al. [33] described under-recognition of restrictive early-onset EDs in Asian children due to differing cultural conceptions of thinness and mental illness. In Chile, Castañeda et al. [32] reported that stigma and misconceptions among healthcare providers delayed specialist referrals, mirroring the Western bias embedded in many diagnostic and training systems. Collectively, these findings highlight the need for culturally sensitive training and public awareness campaigns [66].

The persistence of prolonged DUED despite increasing awareness underscores the need for systematic early-intervention strategies. Evidence from the First Episode Rapid Early Intervention for Eating Disorders (FREED) model in the United Kingdom suggests that structured early-intervention pathways can reduce waiting times and DUED, improve symptom and functional outcomes, and decrease reliance on costly inpatient care and intensive service utilization [67]. Similar approaches, combined with primary-care training [38, 49] and public health campaigns, could replicate these benefits in other settings [68]. Beyond clinical outcomes, economic modelling suggests that enhanced adult AN care pathways combining shorter waits, specialist outpatient care and transition support may reduce healthcare costs by more than 50% [6].

Our study builds upon previous review by incorporating a larger and more diverse body of evidence, including studies published after 2019. The inclusion of studies from Europe, Asia, and Latin America (e.g. [33, 34, 52]), enhances the generalizability of findings across healthcare systems and sociocultural contexts. The inclusion of both pediatric and adult populations permitted direct examination of age-related effects, which emerged as a key moderator. Furthermore, our use of meta-regression represents a methodological advance over prior syntheses, enabling the identification of multiple factors contributing to heterogeneity in DUED.

Several limitations must be acknowledged. First, heterogeneity across studies was extremely high, reflecting substantial variability in populations, settings, and measurement approaches. In particular, definitions of illness onset and treatment initiation varied considerably across studies. While most studies defined illness onset according to the emergence of eating disorder symptoms [16], others used formal diagnosis [35], or alternative indicators, and treatment endpoints ranged from first healthcare contact to treatment initiation. Furthermore, some first-episode studies reported illness duration rather than an explicit measure of DUED, requiring the use of illness duration as a proxy. Together, these differences likely contributed to between-study heterogeneity and limit the comparability of pooled estimates. Many studies also relied on retrospective recall of symptom onset, increasing the potential for recall bias. Although funnel plot asymmetry was observed (Egger’s test *p* < 0.001), the extreme heterogeneity suggests that this more likely reflects small-study effects and genuine between-study variability than publication bias alone.

Second, the current evidence base remains limited in several respects. Despite including studies from Asia and South America, most data still originate from European countries, with only one study from North America (Canada) and no studies from several world regions, limiting the global generalisability of the findings. No studies from the U.S. were identified, despite the high prevalence of EDs there; this may partly reflect financial barriers that have been documented as a significant impediment to accessing ED care in that setting [69]. Although a comprehensive search strategy was employed, including manual reference screening, heterogeneous terminology surrounding treatment delay may have resulted in some relevant studies not being identified. In addition, most included studies were cross-sectional, precluding conclusions regarding the long-term consequences of prolonged DUED or temporal changes in treatment access. Potentially important moderators, including psychiatric and medical comorbidities, socioeconomic factors, and health-system characteristics, could not be examined because they were inconsistently reported. Finally, although the review was guided by a predefined protocol (see Supplementary Materials, item B) and followed PRISMA guidance, the protocol was not prospectively registered, which would have further strengthened the transparency of the review process.

Future studies should prioritize the development of standardized definitions and measurements of DUED and consistent reporting of illness onset, treatment endpoints, and methods of ascertainment to improve comparability across studies. Prospective designs are needed to minimize recall bias and clarify the temporal course of untreated illness. More research is required in underrepresented groups, including males, a broader representation of countries, and individuals with emerging diagnoses such as ARFID. Intervention research should also evaluate the impact of service-level strategies aimed at reducing DUED, such as stepped-care models with strong family engagement to assist with identification of early symptoms, community awareness programs, and digital screening tools, to determine whether the use of specific approaches to reducing delays would translate into improved clinical and functional outcomes.

In conclusion, this systematic review and meta-regression suggest that substantial delays in accessing treatment remain common across ED populations, with evidence that delays may be longer among older individuals and in BN. By identifying factors associated with treatment delays, this study extends previous reviews and provides further insight into barriers to timely care. Reducing DUED should remain an important target for clinical services and public health strategies aimed at improving access to early identification and intervention in EDs.

## Supplementary Information

Below is the link to the electronic supplementary material.

Supplementary Material 1.

## Data Availability

All data analyzed in this study were extracted from previously published articles. The extracted dataset and analytic code are available from the corresponding author upon reasonable request.
