# Supplementary material for: Duration of untreated illness in eating disorders: a comprehensive systematic review and meta-regression
Source: J Eat Disord. 2026 Aug 19;14:245. doi: 10.1186/s40337-026-01731-6 (PMC13679174; doi:10.1186/s40337-026-01731-6)
Supplement: Supplementary file 1 [file 40337_2026_1731_MOESM1_ESM.docx]

**Supplementary materials**

A. **Search strategy**

**OVID Medline**

1	"feeding and eating disorders"/ or anorexia nervosa/ or binge-eating disorder/ or bulimia nervosa/	35084

2	(eating disorder* or disorder* eating or anorexi* or bulimi* or binge eating).tw,kf.	65189

3	1 or 2	71874

4	time-to-treatment/ or treatment delay/	10154

5	((treatment or therapy) adj5 delay*).tw,kf.	45486

6	(duration and untreat*).tw,kf.	9474

7	dued.tw,kf.	17

8	(time adj2 to adj2 (treatment or therapy)).tw,kf.	554

9	(duration adj5 undiagnos*).tw,kf.	36

10	(duration adj3 ill*).tw,kf.	9566

11	((treatment or therapy) adj5 initiation).tw,kf.	53963

12	Undiagnosed Diseases/	199

13	Delayed Diagnosis/	8720

14	or/4-13	130791

15	3 and 14	954

16	exp neoplasms/ or (cancer or neoplasm* or carcinoma).ti.	4275882

17	"feeding and eating disorders"/ or anorexia nervosa/ or binge-eating disorder/ or bulimia nervosa/	35084

18	16 not 17	4275049

19	15 not 18	855

20	exp animals/ not humans/	5231047

21	19 not 20	828

**Embase**

1	eating disorder/ or anorexia nervosa/ or avoidant restrictive food intake disorder/ or binge eating disorder/ or bulimia/ or purging disorder/	62546

2	(eating disorder* or disorder* eating or anorexi* or bulimi* or binge eating).tw.	87905

3	1 or 2	103358

4	(disease duration/ and untreat*.tw.) or *disease duration/	6019

5	time to treatment/	28807

6	therapy delay/	20316

7	((treatment or therapy) adj5 delay*).tw.	70778

8	(duration and untreat*).tw.	16088

9	(duration adj3 ill*).tw.	14801

10	((treatment or therapy) adj5 initiation).tw.	94929

11	dued.tw.	35

12	time to$1 treatment.tw.	9918

13	time to$1 therapy.tw.	390

14	undiagnosed disease/	1106

15	delayed diagnosis/	19934

16	or/4-15	251275

17	3 and 16	1828

18	exp malignant neoplasm/ or (cancer or neoplasm* or carcinoma).ti.	4858335

19	eating disorder/ or anorexia nervosa/ or avoidant restrictive food intake disorder/ or binge eating disorder/ or bulimia/ or purging disorder/	62546

20	18 not 19	4857007

21	17 not 20	1418

22	(exp animals/ or nonhuman/ or animal experiment/ or animal model/) not exp humans/	7459190

23	21 not 22	1375

| **PsycInfo** | | | | |
| --- | --- | --- | --- | --- |
| **#** | **Query** | **Limiters/Expanders** | **Last Run Via** | **Results** |
| S1 | DE "Eating Disorders" OR DE "Anorexia Nervosa" OR DE "Binge Eating Disorder" OR DE "Bulimia" OR DE "Purging (Eating Disorders)" | Expanders - Apply related words; Apply equivalent subjects  Search modes - Boolean/Phrase | Interface - EBSCOhost Research Databases  Search Screen - Advanced Search  Database - APA PsycInfo | 37,128 |
| S2 | TI ( eating disorder* or disorder* eating or anorexi* or binge eating ) OR AB ( eating disorder* or disorder* eating or anorexi* or binge eating ) | Expanders - Apply related words; Apply equivalent subjects  Search modes - Boolean/Phrase | Interface - EBSCOhost Research Databases  Search Screen - Advanced Search  Database - APA PsycInfo | 43,895 |
| S3 | S1 OR S2 | Expanders - Apply related words; Apply equivalent subjects  Search modes - Boolean/Phrase | Interface - EBSCOhost Research Databases  Search Screen - Advanced Search  Database - APA PsycInfo | 48,201 |
| S4 | (treatment or therapy) N5 delay* | Expanders - Apply related words; Apply equivalent subjects  Search modes - Boolean/Phrase | Interface - EBSCOhost Research Databases  Search Screen - Advanced Search  Database - APA PsycInfo | 4,902 |
| S5 | TI ( duration AND untreat* ) OR AB ( duration AND untreat* ) | Expanders - Apply related words; Apply equivalent subjects  Search modes - Boolean/Phrase | Interface - EBSCOhost Research Databases  Search Screen - Advanced Search  Database - APA PsycInfo | 1,755 |
| S6 | dued | Expanders - Apply related words; Apply equivalent subjects  Search modes - Boolean/Phrase | Interface - EBSCOhost Research Databases  Search Screen - Advanced Search  Database - APA PsycInfo | 4 |
| S7 | (time N2 to N2 (treatment or therapy)) | Expanders - Apply related words; Apply equivalent subjects  Search modes - Boolean/Phrase | Interface - EBSCOhost Research Databases  Search Screen - Advanced Search  Database - APA PsycInfo | 21,818 |
| S8 | duration N5 undiagnos* | Expanders - Apply related words; Apply equivalent subjects  Search modes - Boolean/Phrase | Interface - EBSCOhost Research Databases  Search Screen - Advanced Search  Database - APA PsycInfo | 11 |
| S9 | duration N3 ill* | Expanders - Apply related words; Apply equivalent subjects  Search modes - Boolean/Phrase | Interface - EBSCOhost Research Databases  Search Screen - Advanced Search  Database - APA PsycInfo | 5,183 |
| S10 | ((treatment or therapy) N5 initiation) | Expanders - Apply related words; Apply equivalent subjects  Search modes - Boolean/Phrase | Interface - EBSCOhost Research Databases  Search Screen - Advanced Search  Database - APA PsycInfo | 4,768 |
| S11 | undiagnosed disease* | Expanders - Apply related words; Apply equivalent subjects  Search modes - Boolean/Phrase | Interface - EBSCOhost Research Databases  Search Screen - Advanced Search  Database - APA PsycInfo | 59 |
| S12 | delayed N5 diagnosis | Expanders - Apply related words; Apply equivalent subjects  Search modes - Boolean/Phrase | Interface - EBSCOhost Research Databases  Search Screen - Advanced Search  Database - APA PsycInfo | 1,279 |
| S13 | S4 OR S5 OR S6 OR S7 OR S8 OR S9 OR S10 OR S11 OR S12 | Expanders - Apply related words; Apply equivalent subjects  Search modes - Boolean/Phrase | Interface - EBSCOhost Research Databases  Search Screen - Advanced Search  Database - APA PsycInfo | 37,730 |
| S14 | S3 AND S13 | Expanders - Apply related words; Apply equivalent subjects  Search modes - Boolean/Phrase | Interface - EBSCOhost Research Databases  Search Screen - Advanced Search  Database - APA PsycInfo | 1,033 |

| **Cinahl Ultimate** | | | | |
| --- | --- | --- | --- | --- |
| **#** | **Query** | **Limiters/Expanders** | **Last Run Via** | **Results** |
| S1 | (MH "Eating Disorders") OR (MH "Anorexia Nervosa") OR (MH "Binge Eating Disorder") OR (MH "Avoidant Restrictive Food Intake Disorder") OR (MH "Bulimia Nervosa") | Expanders - Apply equivalent subjects  Search modes - Boolean/Phrase | Interface - EBSCOhost Research Databases  Search Screen - Advanced Search  Database - CINAHL Ultimate | 18,005 |
| S2 | TI ( eating disorder* or disorder* eating or anorexi* or binge eating ) OR AB ( eating disorder* or disorder* eating or anorexi* or binge eating ) | Expanders - Apply equivalent subjects  Search modes - Boolean/Phrase | Interface - EBSCOhost Research Databases  Search Screen - Advanced Search  Database - CINAHL Ultimate | 23,676 |
| S3 | S1 OR S2 | Expanders - Apply equivalent subjects  Search modes - Boolean/Phrase | Interface - EBSCOhost Research Databases  Search Screen - Advanced Search  Database - CINAHL Ultimate | 27,908 |
| S4 | (treatment or therapy) N5 delay* | Expanders - Apply equivalent subjects  Search modes - Boolean/Phrase | Interface - EBSCOhost Research Databases  Search Screen - Advanced Search  Database - CINAHL Ultimate | 19,253 |
| S5 | (MH "Turnaround Time") | Expanders - Apply equivalent subjects  Search modes - Boolean/Phrase | Interface - EBSCOhost Research Databases  Search Screen - Advanced Search  Database - CINAHL Ultimate | 645 |
| S6 | dued | Expanders - Apply equivalent subjects  Search modes - Boolean/Phrase | Interface - EBSCOhost Research Databases  Search Screen - Advanced Search  Database - CINAHL Ultimate | 3 |
| S7 | (time N2 to N2 (treatment or therapy)) | Expanders - Apply equivalent subjects  Search modes - Boolean/Phrase | Interface - EBSCOhost Research Databases  Search Screen - Advanced Search  Database - CINAHL Ultimate | 5,878 |
| S8 | (duration N5 undiagnos*). | Expanders - Apply equivalent subjects  Search modes - Boolean/Phrase | Interface - EBSCOhost Research Databases  Search Screen - Advanced Search  Database - CINAHL Ultimate | 18 |
| S9 | TI ( duration and untreat* ) OR AB ( duration and untreat* ) | Expanders - Apply equivalent subjects  Search modes - Boolean/Phrase | Interface - EBSCOhost Research Databases  Search Screen - Advanced Search  Database - CINAHL Ultimate | 1,739 |
| S10 | (duration N3 ill*). | Expanders - Apply equivalent subjects  Search modes - Boolean/Phrase | Interface - EBSCOhost Research Databases  Search Screen - Advanced Search  Database - CINAHL Ultimate | 2,405 |
| S11 | ((treatment or therapy) N5 initiation). | Expanders - Apply equivalent subjects  Search modes - Boolean/Phrase | Interface - EBSCOhost Research Databases  Search Screen - Advanced Search  Database - CINAHL Ultimate | 13,826 |
| S12 | (MH "Undiagnosed Diseases") | Expanders - Apply equivalent subjects  Search modes - Boolean/Phrase | Interface - EBSCOhost Research Databases  Search Screen - Advanced Search  Database - CINAHL Ultimate | 120 |
| S13 | (MH "Diagnosis, Delayed") | Expanders - Apply equivalent subjects  Search modes - Boolean/Phrase | Interface - EBSCOhost Research Databases  Search Screen - Advanced Search  Database - CINAHL Ultimate | 5,462 |
| S14 | (MH "Disease Duration") | Expanders - Apply equivalent subjects  Search modes - Boolean/Phrase | Interface - EBSCOhost Research Databases  Search Screen - Advanced Search  Database - CINAHL Ultimate | 4,945 |
| S15 | S4 OR S5 OR S6 OR S7 OR S8 OR S9 OR S10 OR S11 OR S12 OR S13 OR S14 | Expanders - Apply equivalent subjects  Search modes - Boolean/Phrase | Interface - EBSCOhost Research Databases  Search Screen - Advanced Search  Database - CINAHL Ultimate | 51,013 |
| S16 | S3 AND S15 | Expanders - Apply equivalent subjects  Search modes - Boolean/Phrase | Interface - EBSCOhost Research Databases  Search Screen - Advanced Search  Database - CINAHL Ultimate | 432 |

B. **Study Protocol**

*Duration of untreated illness in eating disorders: a systematic review and metaregression*

**1. Background and rationale**

Eating disorders (EDs) are severe psychiatric conditions associated with substantial morbidity and elevated premature mortality. Early intervention is a key determinant of outcome, yet delays between symptom onset and the start of treatment remain common. The duration of untreated eating disorder (DUED), the interval between illness onset and first treatment, is a potentially modifiable prognostic factor, but estimates vary widely and definitions are inconsistent across studies. This review will estimate the pooled DUED across EDs and examine potential moderators using subgroup and metaregression analyses.

**2. Objectives**

The primary objective will be to estimate the pooled mean DUED (in months) across EDs. Secondary objectives will be to examine variation in DUED by diagnosis, age group, sex, geographic region, decade of data collection and study design, and to explore sources of heterogeneity through meta-regression.

**3. Methods**

*3.1 Reporting and protocol*

The review will be conducted and reported in accordance with the Preferred Reporting Items for Systematic Reviews and Meta-Analyses (PRISMA) guidelines. This protocol will define the eligibility criteria, information sources, search strategy, study selection process, data items, risk-of-bias assessment and planned analyses in advance of data extraction.

*3.2 Eligibility criteria (PICO)*

Studies will be selected according to pre-specified eligibility criteria using the PICO framework:

• Population: Individuals of any age, sex and clinical setting diagnosed with, or meeting criteria for, any eating disorder, including anorexia nervosa (AN), bulimia nervosa (BN), binge-eating disorder (BED), avoidant/restrictive food intake disorder (ARFID), or eating disorder not otherwise specified (EDNOS). Diagnoses based on any established criteria (e.g., DSM, ICD) will be eligible.

• Exposure: DUED, defined as the time elapsed between illness onset and first treatment contact or initiation of care. In first-episode samples, DUED will be operationalised as the duration of illness to date. Where studies of first-episode EDs report duration of illness rather than an explicit DUED measure, duration of illness will be accepted as the closest available proxy, as such studies generally enrol individuals during their first episode.

• Comparator: No comparator group will be required for inclusion.

• Outcomes: The primary outcome will be the mean (or median) DUED in months. Secondary outcomes will include subgroup DUED estimates by diagnosis, age, sex and geographic region.

• Study designs: All observational designs (cross-sectional, cohort, case-control) and interventional study designs will be eligible.

• Language: No language restrictions will be applied; publications in all languages will be eligible.

*3.3 Information sources*

Searches will be conducted from inception to March 2026 in the following databases: PubMed, Embase, PsycINFO and CINAHL. Reference lists of included studies and relevant reviews will be screened manually to identify additional eligible records.

*3.4 Search strategy*

Search strings will combine terms synonymous with eating disorders and with duration of untreated illness / treatment delay. Full, database-specific search strategies will be provided in the Supplementary Materials.

*3.5 Study selection*

Titles and abstracts, followed by full-text screening, will be conducted by three independent authors. Disagreements will be resolved by a senior author (MS) when necessary. The selection process, including the number of records identified, screened, assessed for eligibility and excluded (with reasons), will be summarised in a PRISMA flow diagram.

*3.6 Data extraction and data items*

Following screening, data will be extracted independently by two authors using a pre-specified template. Extracted variables will include the median year and country of data collection, type of ED, DUED, age, gender, sample size, body mass index (BMI) and other psychopathological features. Where more than one definition of DUED is reported within a study, only the definition of symptom onset to treatment will be used, to prevent multiple populations from the same study contributing to the pooled estimate.

*3.7 Risk of bias and quality assessment*

Risk of bias and methodological quality of included studies will be assessed using the National Institutes of Health (NIH) Quality Assessment Tools for observational cohort and cross-sectional studies. Each study will be evaluated across the predefined domains specified in the NIH guidance. Assessments will be conducted independently by two reviewers, with disagreements resolved through discussion and, where necessary, consultation with a third reviewer.

*3.8 Data synthesis and statistical analysis*

All analyses will be conducted in R. Mean DUED and standard deviation (SD) will be pooled using a random-effects meta-analysis with study weights estimated by the Restricted Maximum Likelihood (REML) method. Where DUED is reported as medians, values will be converted to means and SDs using the Wan et al. formulae.

*3.9 Heterogeneity, subgroup analyses and meta-regression*

Heterogeneity will be quantified using the I² statistic, classified as low (75%), and examined via visual inspection of confidence-interval overlap in forest plots. To investigate potential sources of heterogeneity, univariate meta-regression analyses will be conducted for pre-specified moderators with sufficient data (≥10 studies), including mean age, mean BMI, percentage of female participants, year of publication and sample size. Subgroup analyses will be conducted for categorical variables, including country, continent, decade of data collection, age group and study design.

*3.10 Publication bias and sensitivity analyses*

Publication bias will be assessed using Egger’s regression test where the number of studies is ≥10, and via funnel-plot inspection where the number of studies is ≤10. If significant publication bias is detected, a trim-and-fill analysis will be conducted to estimate DUED in the absence of publication bias. Sensitivity analyses will be undertaken to assess the robustness of the pooled estimates.

C. **Supplementary tables and figures**

**Supplementary Table 1: Excluded studies at full-text level**

| **Ref** | **Study bibliography** | **Reason for exclusion** |
| --- | --- | --- |
| 1 | Hyam, Lucy, Yeadon-Ray, Olivia, Richards, Katie, Semple, Amy, Allen, Karina, Owens, Jill, Jackson, Aileen, Semple, Laura, Glennon, Danielle, Di Clemente, Giulia, Griffiths, Jess, Mills, Regan, Schmidt, Ulrike (2024). "FREED instils a bit of hope in the eating disorder community... that things can change.": an investigation of clinician views on implementation facilitators and challenges from the rapid scaling of the First Episode Rapid Early Intervention for Eating Di #journal#, 15(101545006), 1327328, 0.947 | Inappropriate study design or population |
| 2 | Hebebrand, Johannes, Gradl-Dietsch, Gertraud, Peters, Triinu, Correll, Christoph U, Haas, Verena (2024). The Diagnosis and Treatment of Anorexia Nervosa in Childhood and Adolescence. #journal#, 121(5), 164, 0.034 | Inappropriate study design or population |
| 3 | Lebow, Jocelyn, Billings, Marcie, Mattke, Angela, Partain, Paige, Gewirtz O'Brien, Janna, Narr, Cassandra, Breland, Renee, Jacobson, Robert M, Loeb, Katharine, Sim, Leslie (2024). Does embedding pediatric eating disorder treatment in primary care bridge the access gap?. #journal#, 32(3), 283, 0.894 | No data on untreated illness |
| 4 | Nakai, Yoshikatsu, Nin, Kazuko, Noma, Shun'ichi, Teramukai, Satoshi (2024). Onset patterns and clinical features of binge-eating disorder in a Japanese clinical sample. #journal#, 57(3), 740, 0.948 | No data on untreated illness |
| 5 | Iida, Naoko, Ono, Junko, Mizuhara, Yuki, Narumoto, Jin (2023). The subjective assessment of work and social adjustment impairments and associated psychopathologies in Japanese adult female patients with anorexia nervosa. #journal#, 2(4), e151, 0.207 | No data on untreated illness |
| 6 | Vogel, Melina, Gil, Aurora, Galaz, Camila, Urrejola, Pascuala, Lacalle, Lucas, Jara, Raul, Irribarra, Veronica, Letelier, Matias, Costa, Daniela, Espinoza, Gabriela (2023). Virtually Accompanied Eating in the Outpatient Therapy of Anorexia Nervosa. #journal#, 15(17), #Pages#, 0.853 | No data on untreated illness |
| 7 | Hemmingsen, Simone Daugaard, Daugaard, Nicolaj, Sjogren, Magnus, Lichtenstein, Mia Beck, Gudex, Claire, Piil, Frederikke, Stoving, Rene Klinkby (2023). Cognitive Flexibility in Hospitalized Patients with Severe or Extreme Anorexia Nervosa: A Case-Control Study. #journal#, 13(6), #Pages#, 0.884 | No data on untreated illness |
| 8 | Mora-Maltas, Bernat, Lucas, Ignacio, Granero, Roser, Vintro-Alcaraz, Cristina, Miranda-Olivos, Romina, Baenas, Isabel, Sanchez, Isabel, Jimenez-Del Toro, Jessica, Sanchez-Gonzalez, Jessica, Krug, Isabel, Tapia, Javier, Jimenez-Murcia, Susana, Fernandez-Aranda, Fernando (2023). Cognitive flexibility and DSM-5 severity criteria for eating disorders: assessing drive for thinness and duration of illness as alternative severity variables. #journal#, 11(1), 155, 0.998 | No data on untreated illness |
| 9 | Ravanfar, Parsa, Rushmore, R Jarrett, Lyall, Amanda E, Cropley, Vanessa, Makris, Nikos, Desmond, Patricia, Velakoulis, Dennis, Shenton, Martha E, Bush, Ashley I, Rossell, Susan L, Pantelis, Christos, Syeda, Warda T, Phillipou, Andrea (2023). Investigation of brain iron in anorexia nervosa, a quantitative susceptibility mapping study. #journal#, 11(1), 142, 0.149 | No data on untreated illness |
| 10 | Vivarini, Prudence, Jenkins, Zoe M, Castle, David J, Gwee, Karen (2023). Borderline personality disorder symptoms in individuals with eating disorder: Association with severity, psychological distress, and psychosocial function. #journal#, 17(2), 109, 0.949 | No data on untreated illness |
| 11 | Feltner, Cynthia, Peat, Christine, Reddy, Shivani, Riley, Sean, Berkman, Nancy, Middleton, Jennifer Cook, Balio, Casey, Coker-Schwimmer, Manny, Jonas, Daniel E. (2022). Screening for Eating Disorders in Adolescents and Adults: An Evidence Review for the U.S. Preventive Services Task Force #journal#, #volume#(#issue#), #Pages#, 0.81 | No data on untreated illness |
| 12 | Grennan, Laura, Nicula, Maria, Pellegrini, Danielle, Giuliani, Kelly, Crews, Erica, Webb, Cheryl, Gouveia, Maria-Rosa, Loewen, Techiya, Couturier, Jennifer (2022). "I'm not alone": a qualitative report of experiences among parents of children with eating disorders attending virtual parent-led peer support groups. #journal#, 10(1), 195, 0.878 | No data on untreated illness |
| 13 | Takimoto, Yoshiyuki (2022). Indications for involuntary hospitalization for refusal of treatment in severe anorexia nervosa: a survey of physicians and mental health care review board members in Japan. #journal#, 10(1), 176, 0.021 | No data on untreated illness |
| 14 | Berthoz, Sylvie, Maria, Anne-Solene, Ringuenet, Damien, Bourdier, Lena, Nicolas, Isabelle, Blanchet, Corinne, Foulon, Christine, Lavoisy, Guillaume, Godart, Nathalie, Barry, Caroline (2022). Cognitive flexibility and attention to detail in adolescents and adults with severe forms of anorexia nervosa. #journal#, 30(3), 289, 0.856 | No data on untreated illness |
| 15 | Hamilton, Amber, Mitchison, Deborah, Basten, Christopher, Byrne, Susan, Goldstein, Mandy, Hay, Phillipa, Heruc, Gabriella, Thornton, Christopher, Touyz, Stephen (2022). Understanding treatment delay: Perceived barriers preventing treatment-seeking for eating disorders. #journal#, 56(3), 248, 0.844 | No data on untreated illness |
| 16 | Riquin, Elise, Raynal, Agathe, Mattar, Lama, Lalanne, Christophe, Hirot, France, Huas, Caroline, Duclos, Jeanne, Berthoz, Sylvie, Godart, Nathalie (2021). Is the Severity of the Clinical Expression of Anorexia Nervosa Influenced by an Anxiety, Depressive, or Obsessive-Compulsive Comorbidity Over a Lifetime?. #journal#, 12(101545006), 658416, 0.962 | No data on untreated illness |
| 17 | Lin, Jessica A, Jhe, Grace, Vitagliano, Julia A, Milliren, Carly E, Spigel, Rebecca, Woods, Elizabeth R, Forman, Sara F, Richmond, Tracy K (2021). The Association of Malnutrition, illness duration, and pre-morbid weight status with anxiety and depression symptoms in adolescents and young adults with restrictive eating disorders: a cross-sectional study. #journal#, 9(1), 60, 0.912 | No data on untreated illness |
| 18 | Tenconi, Elena, Collantoni, Enrico, Meregalli, Valentina, Bonello, Elisa, Zanetti, Tatiana, Veronese, Angela, Meneguzzo, Paolo, Favaro, Angela (2021). Clinical and Cognitive Functioning Changes After Partial Hospitalization in Patients With Anorexia Nervosa. #journal#, 12(101545006), 653506, 0.816 | No data on untreated illness |
| 19 | Lazare, Kimberly, Mehak, Adrienne, Telner, Deanna (2021). Exploring the primary care experiences of adult patients with eating disorders: a qualitative analysis. #journal#, 29(1), 1, 0.848 | No data on untreated illness |
| 20 | Miranda-Olivos, Romina, Testa, Giulia, Lucas, Ignacio, Sanchez, Isabel, Sanchez-Gonzalez, Jessica, Granero, Roser, Jimenez-Murcia, Susana, Fernandez-Aranda, Fernando (2021). Clinical factors predicting impaired executive functions in eating disorders: The role of illness duration. #journal#, 144(jtj, 0376331), 87, 0.985 | No data on untreated illness |
| 21 | Bemer, Pauline, Di Lodovico, Laura, Haykanush, Ohanyan, Theodon, Helene, Briot, Karine, Carlier, Robert, Dicembre, Marika, Duquesnoy, Maeva, Melchior, Jean-Claude, Hanachi, Mouna (2021). Bone mineral density at extremely low weight in patients with anorexia nervosa. #journal#, 95(3), 423, 0.829 | No data on untreated illness |
| 22 | Eielsen, Hanna Punsvik, Vrabel, KariAnne, Hoffart, Asle, Ro, Oyvind, Rosenvinge, Jan H (2021). The 17-year outcome of 62 adult patients with longstanding eating disorders-A prospective study. #journal#, 54(5), 841, 0.918 | No data on untreated illness |
| 23 | Monteleone, Alessio Maria, Marciello, Francesca, Cascino, Giammarco, Abbate-Daga, Giovanni, Anselmetti, Simona, Baiano, Monica, Balestrieri, Matteo, Barone, Eugenia, Bertelli, Sara, Carpiniello, Bernardo, Castellini, Giovanni, Corrivetti, Giulio, DE Giorgi, Serafino, Favaro, Angela, Gramaglia, Carla, Marzola, Enrica, Meneguzzo, Paolo, Monaco, Francesco, Oriani, Maria Ginevra, Pinna, Federica, Rania, Marianna, Renna, Caterina, Ricca, Valdo, Salvo, Pierandrea, Segura-Garcia, Cristina, Scarabel, Fabiana, Todisco, Patrizia, Volpe, Umberto, Zeppegno, Patrizia, Monteleone, Palmiero (2021). The impact of COVID-19 lockdown and of the following "re-opening" period on specific and general psychopathology in people with Eating Disorders: the emergent role of internalizing symptoms. #journal#, 285(h3v, 7906073), 77, 0.917 | No data on untreated illness |
| 24 | Fernandez-Aranda, Fernando, Treasure, Janet, Paslakis, Georgios, Aguera, Zaida, Gimenez, Monica, Granero, Roser, Sanchez, Isabel, Serrano-Troncoso, Eduardo, Gorwood, Philip, Herpertz-Dahlmann, Beate, Bonin, Eva-Maria, Monteleone, Palmiero, Jimenez-Murcia, Susana (2021). The impact of duration of illness on treatment nonresponse and drop-out: Exploring the relevance of enduring eating disorder concept. #journal#, 29(3), 499, 0.912 | No data on untreated illness |
| 25 | Meule, Adrian, Schrambke, Dominic, Furst Loredo, Artur, Schlegl, Sandra, Naab, Silke, Voderholzer, Ulrich (2021). Inpatient treatment of anorexia nervosa in adolescents: A 1-year follow-up study. #journal#, 29(2), 165, 0.77 | No data on untreated illness |
| 26 | Austin, Amelia, Flynn, Michaela, Richards, Katie, Hodsoll, John, Duarte, Tiago Antunes, Robinson, Paul, Kelly, Jonathan, Schmidt, Ulrike (2021). Duration of untreated eating disorder and relationship to outcomes: A systematic review of the literature. #journal#, 29(3), 329, 0.947 | Inappropriate study design or population |
| 27 | Vanderlinden, Johan, Schoevaerts, Katrien, Simons, Annik, Van Den Eede, Ursula, Bruffaerts, Ronny, Serra, Riccardo, Van Roie, Elke, Vervaet, Myriam, Janssens, Nelle, Vrieze, Elske (2021). Sociodemographic and clinical characteristics of eating disorder patients treated in the specialized residential settings in Belgium. #journal#, 26(2), 475, 0.973 | No data on untreated illness |
| 28 | Christian, Caroline, Williams, Brenna M, Hunt, Rowan A, Wong, Valerie Z, Ernst, Sarah E, Spoor, Samantha P, Vanzhula, Irina A, Tregarthen, Jenna P, Forbush, Kelsie T, Levinson, Cheri A (2021). A network investigation of core symptoms and pathways across duration of illness using a comprehensive cognitive-behavioral model of eating-disorder symptoms. #journal#, 51(5), 815, 0.853 | No data on untreated illness |
| 29 | Wang, Shirley B, Gray, Emily K, Coniglio, Kathryn A, Murray, Helen B, Stone, Melissa, Becker, Kendra R, Thomas, Jennifer J, Eddy, Kamryn T (2021). Cognitive rigidity and heightened attention to detail occur transdiagnostically in adolescents with eating disorders. #journal#, 29(4), 408, 0.936 | No data on untreated illness |
| 30 | Jenkins, Zoe M, Chait, Lior M, Cistullo, Leonardo, Castle, David J (2020). A comparison of eating disorder symptomatology, psychological distress and psychosocial function between early, typical and later onset anorexia nervosa. #journal#, 8(1), 56, 0.973 | No data on untreated illness |
| 31 | Clausen, Loa (2020). Perspectives on Involuntary Treatment of Anorexia Nervosa. #journal#, 11(101545006), 533288, 0.03 | No data on untreated illness |
| 32 | Kwok, Cecilia, Kwok, Victor, Lee, Huei Yen, Tan, Shian Ming (2020). Clinical and socio-demographic features in childhood vs adolescent-onset anorexia nervosa in an Asian population. #journal#, 25(3), 821, 0.864 | No data on untreated illness |
| 33 | Guo, Lei, Wu, Mengting, Zhu, Zhuoying, Zhang, Lei, Peng, Sufang, Li, Wei, Chen, Han, Fernandez-Aranda, Fernando, Chen, Jue (2020). Effectiveness and influencing factors of online education for caregivers of patients with eating disorders during COVID-19 pandemic in China. #journal#, 28(6), 816, 0.942 | No data on untreated illness |
| 34 | Van Alsten, Sarah C, Duncan, Alexis E (2020). Lifetime patterns of comorbidity in eating disorders: An approach using sequence analysis. #journal#, 28(6), 709, 0.972 | No data on untreated illness |
| 35 | Konstantakopoulos, George, Georgantopoulos, Georgios, Gonidakis, Fragiskos, Michopoulos, Ioannis, Stefanatou, Pentagiotissa, David, Anthony S (2020). Development and validation of the schedule for the assessment of insight in eating disorders (SAI-ED). #journal#, 292(qc4, 7911385), 113308, 0.93 | No data on untreated illness |
| 36 | Strand, Mattias, Bulik, Cynthia M, Gustafsson, Sanna A, von Hausswolff-Juhlin, Yvonne, Welch, Elisabeth (2020). Self-admission to inpatient treatment in anorexia nervosa: Impact on healthcare utilization, eating disorder morbidity, and quality of life. #journal#, 53(10), 1685, 0.862 | No data on untreated illness |
| 37 | Mensinger, Janell L, Granche, Janeway L, Cox, Shelbi A, Henretty, Jennifer R (2020). Sexual and gender minority individuals report higher rates of abuse and more severe eating disorder symptoms than cisgender heterosexual individuals at admission to eating disorder treatment. #journal#, 53(4), 541, 0.929 | No data on untreated illness |
| 38 | Li, Xiao, Liu, Xiaowei, Wang, Yu, Li, Lingfei, Zheng, Linli, Liu, Yaya, Ma, Jing, Zhang, Lan (2020). Altered regional gray matter volume in Chinese female patients with bulimia nervosa. #journal#, 20(1), 94, 0.836 | No data on untreated illness |
| 39 | Ambwani, Suman, Cardi, Valentina, Albano, Gaia, Cao, Li, Crosby, Ross D, Macdonald, Pamela, Schmidt, Ulrike, Treasure, Janet (2020). A multicenter audit of outpatient care for adult anorexia nervosa: Symptom trajectory, service use, and evidence in support of "early stage" versus "severe and enduring" classification. #journal#, 53(8), 1337, 0.865 | No data on untreated illness |
| 40 | Rizk, Melissa, Mattar, Lama, Kern, Laurence, Berthoz, Sylvie, Duclos, Jeanne, Viltart, Odile, Godart, Nathalie (2020). Physical Activity in Eating Disorders: A Systematic Review. #journal#, 12(1), #Pages#, 0.919 | No data on untreated illness |
| 41 | Fukutomi, Akira, Austin, Amelia, McClelland, Jessica, Brown, Amy, Glennon, Danielle, Mountford, Victoria, Grant, Nina, Allen, Karina, Schmidt, Ulrike (2020). First episode rapid early intervention for eating disorders: A two-year follow-up. #journal#, 14(1), 137, 0.898 | Inappropriate study design or population |
| 42 | Monteleone, Alessio Maria, Mereu, Alberta, Cascino, Giammarco, Criscuolo, Michela, Castiglioni, Maria Chiara, Pellegrino, Francesca, Patriciello, Giuseppina, Ruzzi, Valeria, Monteleone, Palmiero, Vicari, Stefano, Zanna, Valeria (2019). Re-conceptualization of anorexia nervosa psychopathology: A network analysis study in adolescents with short duration of the illness. #journal#, 52(11), 1263, 0.884 | No data on untreated illness |
| 43 | Volpe, Umberto, Monteleone, Alessio M, Ricca, Valdo, Corsi, Elisa, Favaro, Angela, Santonastaso, Paolo, De Giorgi, Serafino, Renna, Caterina, Abbate Daga, Giovanni, Amianto, Federico, Balestrieri, Matteo, Luxardi, Gian Luigi, Clerici, Massimo, Alamia, Alberto, Segura-Garcia, Cristina, Rania, Marianna, Monteleone, Palmiero, Maj, Mario (2019). Pathways to specialist care for eating disorders: An Italian multicentre study. #journal#, 27(3), 274, 0.892 | No data on untreated illness |
| 44 | Berner, Laura A, Wang, Zhishun, Stefan, Mihaela, Lee, Seonjoo, Huo, Zhiyong, Cyr, Marilyn, Marsh, Rachel (2019). Subcortical Shape Abnormalities in Bulimia Nervosa. #journal#, 4(12), 1070, 0.292 | No data on untreated illness |
| 45 | Lieberman, Melissa, Houser, Melissa E, Voyer, Anne-Pier, Grady, Shelley, Katzman, Debra K (2019). Children with avoidant/restrictive food intake disorder and anorexia nervosa in a tertiary care pediatric eating disorder program: A comparative study. #journal#, 52(3), 239, 0.818 | No data on untreated illness |
| 46 | Perko, Victoria L, Forbush, Kelsie T, Siew, Cynthia S Q, Tregarthen, Jenna P (2019). Application of network analysis to investigate sex differences in interactive systems of eating-disorder psychopathology. #journal#, 52(12), 1343, 0.883 | No data on untreated illness |
| 47 | Takakura, Shu, Aso, Chie Suzuyama, Toda, Kenta, Hata, Tomokazu, Yamashita, Makoto, Sudo, Nobuyuki (2019). Physical and psychological aspects of anorexia nervosa based on duration of illness: a cross-sectional study. #journal#, 13(101286572), 32, 0.933 | No data on untreated illness |
| 48 | Kastner, Denise, Buchholz, Ines, Weigel, Angelika, Brunner, Romuald, Voderholzer, Ulrich, Gumz, Antje, Lowe, Bernd (2019). Facilitators and barriers in anorexia nervosa treatment initiation (FABIANA): study protocol for a mixed-methods and multicentre study. #journal#, 5(6), e92, 0.918 | No data on untreated illness |
| 49 | Andersen, Susanne B, Lindgreen, Pil, Rokkedal, Kristian, Clausen, Loa (2018). Grasping the weight cut-off for anorexia nervosa in children and adolescents. #journal#, 51(12), 1346, 0.937 | No data on untreated illness |
| 50 | Strand, Mattias, von Hausswolff-Juhlin, Yvonne, Welch, Elisabeth (2018). [ARFID: food restriction without fear of weight gain]. #journal#, 115(l0n, 0027707), #Pages#, 0.854 | No data on untreated illness |
| 51 | Miyawaki, Dai, Goto, Ayako, Harada, Tomoko, Yamauchi, Tsuneo, Iwakura, Yoshihiro, Terakawa, Hiroki, Hirai, Kaoru, Miki, Yusuke, Harima, Yuji, Inoue, Koki (2018). High prevalence of shoplifting in patients with eating disorders. #journal#, 23(6), 761, 0.934 | No data on untreated illness |
| 52 | Kouidrat, Youssef, Amad, Ali, Stubbs, Brendon, Louhou, Rufin, Renard, Nathalie, Diouf, Momar, Lalau, Jean-Daniel, Loas, Gwenole (2018). Disordered eating behaviors as a potential obesogenic factor in schizophrenia. #journal#, 269(qc4, 7911385), 450, 0.878 | No data on untreated illness |
| 53 | Murray, Stuart B, Rand-Giovannetti, Devin, Griffiths, Scott, Nagata, Jason M (2018). Locating the mechanisms of therapeutic agency in family-based treatment for adolescent anorexia nervosa: A pilot study of clinician/researcher perspectives. #journal#, 26(5), 477, 0.773 | No data on untreated illness |
| 54 | Brown, Tiffany A, Cusack, Anne, Anderson, Leslie K, Trim, Julie, Nakamura, Tiffany, Trunko, Mary Ellen, Kaye, Walter H (2018). Efficacy of a partial hospital programme for adults with eating disorders. #journal#, 26(3), 241, 0.863 | No data on untreated illness |
| 55 | Chapa, Danielle A N, Bohrer, Brittany K, Forbush, Kelsie T (2018). Is the diagnostic threshold for bulimia nervosa clinically meaningful?. #journal#, 28(101090048), 16, 0.873 | No data on untreated illness |
| 56 | Jenkins, Paul E, Price, Tom (2018). Eating pathology in midlife women: Similar or different to younger counterparts?. #journal#, 51(1), 3, 0.91 | No data on untreated illness |
| 57 | Forsen Mantilla, Emma, Clinton, David, Birgegard, Andreas (2018). Insidious: The relationship patients have with their eating disorders and its impact on symptoms, duration of illness, and self-image. #journal#, 91(3), 302, 0.911 | No data on untreated illness |
| 58 | Lehmann, Celine S, Hofmann, Tobias, Elbelt, Ulf, Rose, Matthias, Correll, Christoph U, Stengel, Andreas, Haas, Verena (2018). The Role of Objectively Measured, Altered Physical Activity Patterns for Body Mass Index Change during Inpatient Treatment in Female Patients with Anorexia Nervosa. #journal#, 7(9), #Pages#, 0.801 | No data on untreated illness |
| 59 | Brown, Andrea, Jenkinson, Richard, Coakes, Julia, Cockfield, Annette, O'Brien, Tish, Hall, Louise (2018). Pathways to Recovery: development and evaluation of a cognitive-behavioural therapy in-patient treatment programme for adults with anorexia nervosa. #journal#, 42(3), 95, 0.803 | No data on untreated illness |
| 60 | Keshen, Aaron, Helson, Thomas, Town, Joel, Warren, Karly (2017). Self-efficacy as a predictor of treatment outcome in an outpatient eating disorder program. #journal#, 25(5), 406, 0.844 | No data on untreated illness |
| 61 | Gianini, Loren, Roberto, Christina A, Attia, Evelyn, Walsh, B Timothy, Thomas, Jennifer J, Eddy, Kamryn T, Grilo, Carlos M, Weigel, Thomas, Sysko, Robyn (2017). Mild, moderate, meaningful? Examining the psychological and functioning correlates of DSM-5 eating disorder severity specifiers. #journal#, 50(8), 906, 0.895 | No data on untreated illness |
| 62 | Foerde, Karin, Steinglass, Joanna E (2017). Decreased feedback learning in anorexia nervosa persists after weight restoration. #journal#, 50(4), 415, 0.794 | No data on untreated illness |
| 63 | Nakai, Yoshikatsu, Nin, Kazuko, Noma, Shun'ichi, Hamagaki, Seiji, Takagi, Ryuro, Teramukai, Satoshi, Wonderlich, Stephen A (2017). Clinical presentation and outcome of avoidant/restrictive food intake disorder in a Japanese sample. #journal#, 24(101090048), 49, 0.854 | No data on untreated illness |
| 64 | Rosner, Anne, Juniak, Izabela, van Noort, Betteke Maria, Pfeiffer, Ernst, Lehmkuhl, Ulrike, Kappel, Viola (2017). Cognitive Flexibility in Juvenile Anorexia Nervosain Relation to Comorbid Symptoms of Depression, Obsessive Compulsive Symptoms and Duration of Illness. #journal#, 45(5), 371, 0.877 | No data on untreated illness |
| 65 | Calugi, Simona, El Ghoch, Marwan, Dalle Grave, Riccardo (2017). Intensive enhanced cognitive behavioural therapy for severe and enduring anorexia nervosa: A longitudinal outcome study. #journal#, 89(9kp, 0372477), 41, 0.844 | No data on untreated illness |
| 66 | Vo, Megen, Accurso, Erin C, Goldschmidt, Andrea B, Le Grange, Daniel (2017). The Impact of DSM-5 on Eating Disorder Diagnoses. #journal#, 50(5), 578, 0.882 | No data on untreated illness |
| 67 | Davidsen, Annika Helgadottir, Hoyt, William T, Poulsen, Stig, Waaddegaard, Mette, Lau, Marianne (2017). Eating disorder severity and functional impairment: moderating effects of illness duration in a clinical sample. #journal#, 22(3), 499, 0.985 | No data on untreated illness |
| 68 | Nakai, Yoshikatsu, Nin, Kazuko, Noma, Shun'ichi, Teramukai, Satoshi, Wonderlich, Stephen A (2016). Characteristics of Avoidant/Restrictive Food Intake Disorder in a Cohort of Adult Patients. #journal#, 24(6), 528, 0.938 | No data on untreated illness |
| 69 | Lavagnino, Luca, Amianto, Federico, Mwangi, Benson, D'Agata, Federico, Spalatro, Angela, Zunta Soares, Giovana B, Daga, Giovanni Abbate, Mortara, Paolo, Fassino, Secondo, Soares, Jair C (2016). The relationship between cortical thickness and body mass index differs between women with anorexia nervosa and healthy controls. #journal#, 248(101723001), 105, 0.829 | No data on untreated illness |
| 70 | Seitz, Jochen, Walter, Martin, Mainz, Verena, Herpertz-Dahlmann, Beate, Konrad, Kerstin, von Polier, Georg (2015). Brain volume reduction predicts weight development in adolescent patients with anorexia nervosa. #journal#, 68(jtj, 0376331), 228, 0.755 | No data on untreated illness |
| 71 | Treasure, Janet, Stein, Daniel, Maguire, Sarah (2015). Has the time come for a staging model to map the course of eating disorders from high risk to severe enduring illness? An examination of the evidence. #journal#, 9(3), 173, 0.819 | No data on untreated illness |
| 72 | Shu, Chloe Y, Limburg, Karina, Harris, Chris, McCormack, Julie, Hoiles, Kimberley J, Hamilton, Matthew J, Watson, Hunna J (2015). Clinical presentation of eating disorders in young males at a tertiary setting. #journal#, 3(101610672), 39, 0.125 | No data on untreated illness |
| 73 | Gumz, Antje, Uhlenbusch, Natalie, Weigel, Angelika, Wegscheider, Karl, Romer, Georg, Lowe, Bernd (2014). Decreasing the duration of untreated illness for individuals with anorexia nervosa: study protocol of the evaluation of a systemic public health intervention at community level. #journal#, 14(100968559), 300, 0.898 | No data on untreated illness |
| 74 | von Lojewski, Astrid, Abraham, Suzanne (2014). Personality factors and eating disorders: self-uncertainty. #journal#, 15(1), 106, 0.896 | No data on untreated illness |
| 75 | Clausen, Loa, Jones, Allan (2014). A systematic review of the frequency, duration, type and effect of involuntary treatment for people with anorexia nervosa, and an analysis of patient characteristics. #journal#, 2(1), 29, 0.095 | No data on untreated illness |
| 76 | Masheb, Robin M, White, Marney A, Grilo, Carlos M (2013). Substantial weight gains are common prior to treatment-seeking in obese patients with binge eating disorder. #journal#, 54(7), 880, 0.083 | No data on untreated illness |
| 77 | Ruwaard, Jeroen, Lange, Alfred, Broeksteeg, Janneke, Renteria-Agirre, Aitziber, Schrieken, Bart, Dolan, Conor V, Emmelkamp, Paul (2013). Online cognitive-behavioural treatment of bulimic symptoms: a randomized controlled trial. #journal#, 20(4), 308, 0.772 | No data on untreated illness |
| 78 | Pinzon, Vanessa Dentzien, Turkiewicz, Gizela, Monteiro, Denise Oliveira, Koritar, Priscila, Fleitlich-Bilyk, Bacy (2013). Who are the children and adolescent patients of a national referral service of eating disorders in Brazil?: a cross-sectional study of a clinical sample. #journal#, 35(3), 221, 0.966 | No data on untreated illness |
| 79 | DeJong, Hannah, Oldershaw, Anna, Sternheim, Lot, Samarawickrema, Nelum, Kenyon, Martha D, Broadbent, Hannah, Lavender, Anna, Startup, Helen, Treasure, Janet, Schmidt, Ulrike (2013). Quality of life in anorexia nervosa, bulimia nervosa and eating disorder not-otherwise-specified. #journal#, 1(101610672), 43, 0.898 | No data on untreated illness |
| 80 | Forman, Sara F, Grodin, Leah F, Graham, Dionne A, Sylvester, Cara J, Rosen, David S, Kapphahn, Cynthia J, Callahan, S Todd, Sigel, Eric J, Bravender, Terrill, Peebles, Rebecka, Romano, Mary, Rome, Ellen S, Fisher, Martin, Malizio, Joan B, Mammel, Kathleen A, Hergenroeder, Albert C, Buckelew, Sara M, Golden, Neville H, Woods, Elizabeth R (2011). An eleven site national quality improvement evaluation of adolescent medicine-based eating disorder programs: predictors of weight outcomes at one year and risk adjustment analyses. #journal#, 49(6), 594, 0.93 | No data on untreated illness |
| 81 | Monteleone, Palmiero, Di Genio, Monica, Monteleone, Alessio Maria, Di Filippo, Carmela, Maj, Mario (2011). Investigation of factors associated to crossover from anorexia nervosa restricting type (ANR) and anorexia nervosa binge-purging type (ANBP) to bulimia nervosa and comparison of bulimia nervosa patients with or without previous ANR or ANBP. #journal#, 52(1), 56, 0.838 | No data on untreated illness |
| 82 | Dostalova, Ivana, Kavalkova, Petra, Papezova, Hana, Domluvilova, Daniela, Zikan, Vit, Haluzik, Martin (2010). Association of macrophage inhibitory cytokine-1 with nutritional status, body composition and bone mineral density in patients with anorexia nervosa: the influence of partial realimentation. #journal#, 7(101231644), 34, 0.799 | No data on untreated illness |
| 83 | Bamford, Bryony, Sly, Richard (2010). Exploring quality of life in the eating disorders. #journal#, 18(2), 147, 0.927 | No data on untreated illness |
| 84 | Fennig, Silvana, Hadas, Arie (2010). Suicidal behavior and depression in adolescents with eating disorders. #journal#, 64(1), 32, 0.125 | No data on untreated illness |
| 85 | Nishimura, Hiroki, Komaki, Gen, Ando, Tetsuya, Nakahara, Toshihiro, Oka, Takakazu, Kawai, Keisuke, Nagata, Toshihiko, Nishizono, Aya, Okamoto, Yuri, Okabe, Kenjiro, Koide, Masanori, Yamaguchi, Chikara, Saito, Satoshi, Ohkuma, Kazuyoshi, Nagata, Katsutaro, Naruo, Tetsuro, Takii, Masato, Kiriike, Nobuo, Ishikawa, Toshio (2008). Psychological and weight-related characteristics of patients with anorexia nervosa-restricting type who later develop bulimia nervosa. #journal#, 2(101286572), 5, 0.899 | No data on untreated illness |
| 86 | Misra, Madhusmita (2008). Long-term skeletal effects of eating disorders with onset in adolescence. #journal#, 1135(5nm, 7506858), 212, 0.817 | No data on untreated illness |
| 87 | Graap, Holmer, Bleich, Stefan, Herbst, Florian, Scherzinger, Charlotte, Trostmann, Yvonne, Wancata, Johannes, de Zwaan, Martina (2008). The needs of carers: a comparison between eating disorders and schizophrenia. #journal#, 43(10), 800, 0.868 | No data on untreated illness |
| 88 | Casasnovas, C, Fernandez-Aranda, F, Granero, R, Krug, I, Jimenez-Murcia, S, Bulik, C M, Vallejo-Ruiloba, J (2007). Motivation to change in eating disorders: clinical and therapeutic implications. #journal#, 15(6), 449, 0.916 | No data on untreated illness |
| 89 | Perkins, S, Schmidt, U, Eisler, I, Treasure, J, Berelowitz, M, Dodge, E, Frost, S, Jenkins, M, Johnson-Sabine, E, Keville, S, Murphy, R, Robinson, P, Winn, S, Yi, I (2007). Motivation to change in recent onset and long-standing bulimia nervosa: are there differences?. #journal#, 12(2), 61, 0.893 | No data on untreated illness |
| 90 | Pope, Harrison G Jr, Lalonde, Justine K, Pindyck, Lindsay J, Walsh, Timothy, Bulik, Cynthia M, Crow, Scott J, McElroy, Susan L, Rosenthal, Norman, Hudson, James I (2006). Binge eating disorder: a stable syndrome. #journal#, 163(12), 2181, 0.892 | No data on untreated illness |
| 91 | Viglione, Valentina, Muratori, Filippo, Maestro, Sandra, Brunori, Elena, Picchi, Lara (2006). Denial of symptoms and psychopathology in adolescent anorexia nervosa. #journal#, 39(5), 255, 0.879 | No data on untreated illness |
| 92 | Takakura, S, Nozaki, T, Nomura, Y, Koreeda, C, Urabe, H, Kawai, K, Takii, M, Kubo, C (2006). Factors related to renal dysfunction in patients with anorexia nervosa. #journal#, 11(2), 73, 0.854 | No data on untreated illness |
| 93 | Nozaki, T, Takao, M, Takakura, S, Koreeda-Arimura, C, Ishido, K, Yamada, Y, Kawai, K, Takii, M, Kubo, C (2006). Psychopathological features of patients with prolonged anorexia nervosa as assessed by the Minnesota Multiphasic Personality Inventory. #journal#, 11(2), 59, 0.892 | No data on untreated illness |
| 94 | Hueg, Anne, Resch, Franz, Haffner, Johann, Poustka, Luise, Parzer, Peter, Brunner, Romuald (2006). [Temperament and character profiles of female adolescent patients with anorexia and bulimia nervosa]. #journal#, 34(2), 127, 0.908 | No data on untreated illness |
| 95 | McDermott, Brett, Forbes, David, Harris, Chris, McCormack, Julie, Gibbon, Peter (2006). Non-eating disorders psychopathology in children and adolescents with eating disorders: implications for malnutrition and symptom severity. #journal#, 60(3), 257, 0.992 | No data on untreated illness |
| 96 | Shimizu, Toshiaki, Satoh, Yayoi, Kaneko, Noritsugu, Suzuki, Mitsuyoshi, Lee, Tsubasa, Tanaka, Kyoko, Iijima, Megumi, Yamashiro, Yuichiro (2005). Factors involved in the regulation of plasma leptin levels in children and adolescents with anorexia nervosa. #journal#, 47(2), 154, 0.746 | No data on untreated illness |
| 97 | Ogawa, Asao, Mizuta, Ichiro, Fukunaga, Tomoko, Takeuchi, Naoko, Honaga, Eiko, Sugita, Yoshiro, Mikami, Akira, Inoue, Yoichi, Takeda, Masatoshi (2004). Electrogastrography abnormality in eating disorders. #journal#, 58(3), 300, 0.863 | No data on untreated illness |
| 98 | Di Pietro, G, Valoroso, L, Fichele, M, Bruno, C, Sorge, F (2002). What happens to eating disorder outpatients who withdrew from therapy?. #journal#, 7(4), 298, 0.897 | No data on untreated illness |
| 99 | Treasure, J, Murphy, T, Szmukler, G, Todd, G, Gavan, K, Joyce, J (2001). The experience of caregiving for severe mental illness: a comparison between anorexia nervosa and psychosis. #journal#, 36(7), 343, 0.819 | No data on untreated illness |
| 100 | Kiriike, N, Nagata, T, Matsunaga, H, Tobitan, W, Nishiura, T (1998). Single and married patients with eating disorders. #journal#, 52 Suppl(cfs, 9513551), S306, 0.944 | No data on untreated illness |
| 101 | Kruger, S, McVey, G, Kennedy, S H (1998). The changing profile of anorexia nervosa at the Toronto Programme for Eating Disorders. #journal#, 45(6), 533, 0.836 | No data on untreated illness |
| 102 | Tiller, J M, Sloane, G, Schmidt, U, Troop, N, Power, M, Treasure, J L (1997). Social support in patients with anorexia nervosa and bulimia nervosa. #journal#, 21(1), 31, 0.911 | No data on untreated illness |
| 103 | Kiriike, N, Nagata, T, Matsunaga, H, Tobitani, W, Nishiura, T (1996). Married patients with eating disorders in Japan. #journal#, 94(6), 428, 0.935 | No data on untreated illness |
| 104 | Clinton, D N, Glant, R (1992). The eating disorders spectrum of DSM-III-R. Clinical features and psychosocial concomitants of 86 consecutive cases from a Swedish urban catchment area. #journal#, 180(4), 244, 0.864 | No data on untreated illness |
| 105 | Pearson, D A, Pumariega, A J, Seilheimer, D K (1991). The development of psychiatric symptomatology in patients with cystic fibrosis. #journal#, 30(2), 290, 0.892 | No data on untreated illness |
| 106 | Laessle, R G, Krieg, J C, Fichter, M M, Pirke, K M (1989). Cerebral atrophy and vigilance performance in patients with anorexia nervosa and bulimia nervosa. #journal#, 21(4), 187, 0.913 | No data on untreated illness |
| 107 | Hsu, L K, Holder, D (1986). Bulimia nervosa: treatment and short-term outcome. #journal#, 16(1), 65, 0.832 | No data on untreated illness |
| 108 | Casper, R C, Offer, D, Ostrov, E (1981). The self-image of adolescents with acute anorexia nervosa. #journal#, 98(4), 656, 0.885 | No data on untreated illness |
| 109 | Cremerius, J (1965). [On the prognosis of anorexia nervosa (13 catamneses of 15 to 18 years duration of psychotherapeutically untreated cases)]. #journal#, 207(4), 378, 0.885 | No data on untreated illness |
| 110 | McDaid D., Treasure J., Fernandez-Aranda F., Herpertz-Dahlmann B., Quoidbach V., Dickson S., Gorwood P. (2024). Quantifying the economic value of earlier and enhanced management of Anorexia Nervosa for adults in England, Germany and Spain: improving the care pathway #journal#, #volume#((McDaid) Care Policy and Evaluation Centre, Department of Health Policy, London School of Economics and Political Science, London, United Kingdom(Treasure) Centre for Research in Eating and Weight Disorders (CREW), Institute of Psychiatry, Psychology and), #Pages#, 0.779 | No data on untreated illness |
| 111 | Cohensedgh O., Uniacke B. (2023). 1.40 Sexual and Gender Identity Development in Adolescents With Anorexia Nervosa #journal#, 62(10 Supplement), S168, 0.898 | No data on untreated illness |
| 112 | Daneshdoost S., Adams K., Cooper M., Timko A., Weaver L., Seidman C., Peebles R., Singh A. (2023). DEMYSTIFYING THE COEXISTENCE OF CELIAC DISEASE AND EATING DISORDERS #journal#, 77(1 Supplement 1), S77, 0.951 | No data on untreated illness |
| 113 | Daugelat M.-C., Pruccoli J., Schag K., Giel K.E. (2023). Barriers and facilitators affecting treatment uptake behaviours for patients with eating disorders: A systematic review synthesising patient, caregiver and clinician perspectives #journal#, 31(6), 752, 0.821 | No data on untreated illness |
| 114 | Herrmann K., Kaluscha R., Liebert A., Spohrs J., Gundel H., von Wietersheim J. (2022). First onset of treatment of patients with eating disorders and treatment course: Results of data from a German health insurance company #journal#, 30(6), 787, 0.832 | No data on untreated illness |
| 115 | Breithaupt L., Lyall A., Petterway F., Carrington H., Holsen L., Plessow F., Chen Y., Thomas J., Misa M., Lawson E., Eddy K. (2022). Shape and Volume Alterations in Habit-Centered Basal Ganglia Structures Present in Atypical Anorexia Nervosa #journal#, 47(Supplement 1), 167, 0.169 | No data on untreated illness |
| 116 | Pruccoli J., Pelusi M., Romagnoli G., Parmeggiani A. (2022). The impact of nasogastric tube feeding on Drive for Thinness and Body Dissatisfaction in children and adolescents with Anorexia Nervosa #journal#, 65(Supplement 1), S384, 0.871 | Conference abstract |
| 117 | Barone E., Marciello F., Cascino G., Abbate-Daga G., Anselmetti S., Baiano M., Balestrieri M., Bertelli S., Carpiniello B., Castellini G., Corrivetti G., De Giorgi S., Favaro A., Gramaglia C., Marzola E., Monaco F., Oriani M.G., Federica P., Rania M., Renna C., Ricca V., Salvo P., Segura-Garcia C., Scarabel F., Todisco P., Volpe U., Zeppegno P., Monteleone P., Monteleone A.M. (2021). COVID-19 pandemic and eating disorders: What impact on specific and general psychopathology? #journal#, 64(Supplement 1), S114, 0.954 | No data on untreated illness |
| 118 | Pruccoli J., La Tempa A., Gualandi P., Francia V., Malaspina E., Moscano F., Rossi F., Sacrato L., Rucci P., Parmeggiani A. (2021). First- and second-generation immigrant adolescents in Italy with anorexia nervosa, need for treatment and clinical outcomes #journal#, 29(6), E24, 0.092 | No data on untreated illness |
| 119 | Pelusi M., Pruccoli J., Romagnoli G., Parmeggiani A. (2021). Predictors of weight restoration for nasogastric tube feeding in the treatment of children and adolescents with anorexia nervosa: an observational study #journal#, 46((Pelusi, Pruccoli, Parmeggiani) IRCCS Istituto Delle Scienze Neurologiche di Bologna, Regional Centre for Feeding and Eating Disorders in Children and Adolescents, Italy(Pelusi, Pruccoli, Romagnoli, Parmeggiani) Department of Medical and Surgical Sciences), S726, 0.868 | Inappropriate study design or population |
| 120 | Jonathan S., Ghassan W. (2020). Restrictive Eating Symptoms May Persist in Children & Adolescents With Treated IBD: Case Series #journal#, 115(Supplement), S14, 0.955 | Inappropriate study design or population |
| 121 | Cascino G., Mereu A., Zanna V., Monteleone P., Vicari S., Monteleone A.M. (2020). The validity of the 5th BMI percentile as weight cut-off for anorexia nervosa in children and adolescents: No evidence from a psychopathology investigation #journal#, 63(Supplement 1), S41, 0.954 | No data on untreated illness |
| 122 | Ruiz Guerrero F., Losa Mujika E., Gandara Gutierrez C., Benito Gonzalez P., Calcedo Giraldo G., Gonzalez Gomez J., Gomez Del Barrio A. (2020). Reduction of duration untreated eating disorder (DUed) in a sample of patients with an eating disorder #journal#, 63(Supplement 1), S381, 0.833 | No data on untreated illness |
| 123 | Grau A., Magallon-Neri E., Faus G., Feixas G. (2019). Cognitive impairment in eating disorder patients of short and long-term duration: A case-control study #journal#, 15((Grau, Faus) ITA Mental Health Specialist, Madrid, Spain(Grau, Faus) ITA Foundation, Barcelona, Spain(Magallon-Neri, Feixas) Department of Clinical Psychology and Psychobiology, Faculty of Psychology, University of Barcelona, Barcelona, Spain(Magallon-Ner), 1329, 0.952 | No data on untreated illness |
| 124 | Chandran M., Hao Y. (2019). Willthere be a fracture in their future? Bone mineral density findings in young southeast asian women with anorexia nervosa #journal#, 30(SUPPL 2), S471, 0.811 | No data on untreated illness |
| 125 | Kinasz K.R., Gorrell S., Trainor C., Le Grange D. (2019). 1.8 ANOREXIA NERVOSA VERSUS AVOIDANT/RESTRICTIVE FOOD INTAKE DISORDER (ARFID) IN TREATMENT-SEEKING ADOLESCENTS: HOW DO THESE POPULATIONS DIFFER? #journal#, 58(10 Supplement), S149, 0.914 | No data on untreated illness |
| 126 | Rastogi R., Nahra A., Neifert N., Sieke E.H., Issac V., Rome E.S. (2019). Wrist Size Among Adolescents With Restrictive Eating Disorders #journal#, 64(2 Supplement), S54, 0.862 | No data on untreated illness |
| 127 | Birchwood M. (2018). Youth space and youth mental health #journal#, 48(Supplement 1), S7, 0.922 | Inappropriate study design or population |
| 128 | Matthews A., Peterson C., Mitan L. (2018). Characteristics and weight related outcomes in medically hospitalized youth with anorexia nervosa #journal#, 62(2 Supplement 1), S102, 0.067 | No data on untreated illness |
| 129 | Forsen Mantilla E., Birgegard A. (2017). Insidious: The relationship between patients and their eating disorders and its impact on ED symptoms, illness duration and self-image #journal#, 41(Supplement 1), S552, 0.976 | No data on untreated illness |
| 130 | Zerwas S., Yilmaz Z., Thornton L., Eusden J., Duncan L., Micali N., Huckins L., Baker J., Munn-Chernoff M., Crowley J.J., Zeggini E., Breen G., Bulik C. (2017). Anorexia nervosa polygenic risk score: Preliminary analyses #journal#, 27(Supplement 2), S320, 0.039 | No data on untreated illness |
| 131 | O'Connor C., O'Hara L., McNamara N., McNicholas F. (2015). EATING DISORDER LITERACY AND STIGMATISATION: A SURVEY OF IRISH ADOLESCENTS #journal#, 69(Supplement 1), A69, 0.873 | No data on untreated illness |
| 132 | Can as L., Serrano E., Carulla M., Sanchez B., San L. (2015). Systematic review of avoidant/restrictive food intake disorder in children and adolescents #journal#, 24(1 SUPPL. 1), S167, 0.835 | No data on untreated illness |
| 133 | Simion A., Crasan A., Calin D., Muscalu M., Macovei S. (2014). Challenges in treating children and adolescents with antipsychotic agents #journal#, 24(SUPPL. 2), S518, 0.717 | No data on untreated illness |
| 134 | Sebastian M.R., Hergenroeder A. (2014). Does rate of weight gain during hospitalization predict readmission in adolescents with eating disorders? #journal#, 54(2 SUPPL. 1), S78, 0.086 | No data on untreated illness |
| 135 | Abbate-Daga G., Amianto F., Delsedime N., De-Bacco C., Fassino S. (2013). Resistance to treatment in eating disorders: A critical challenge #journal#, 13((Abbate-Daga, Amianto, Delsedime, De-Bacco, Fassino) Eating Disorders Center for Treatment and Research, Department of Neuroscience, University of Turin, Turin, Italy), 294, 0.064 | No data on untreated illness |
| 136 | Franceschilli L., Iacoangeli F., Staar F., De Luca E., Giorgi F., Raniolo M., Perrone F., Gaspari L.A., Sileri P.P. (2013). Defaecatory disorders in patients with anorexia nervosa #journal#, 17(1), 141, 0.922 | No data on untreated illness |
| 137 | Forman S.F., McKenzie N.M., Monge M.C., Mammel K.A., Kapphahn C.J., Callahan S.T., Rome E.S., Rosen D.S., Hehn R.S., Carlson J.L., Romano M.E., Malizio J.B., Rouse M.R., Graham D.A., Jay M.S., Hergenroeder A.C., Fisher M.M., Golden N.H., Woods E.R. (2013). Report from the national eating disorder quality improvement collaborative of ten adolescent medicine-based eating disorder programs: Population description and predictors of weight outcome at one year #journal#, 52(2 SUPPL. 1), S37, 0.87 | No data on untreated illness |
| 138 | Fisher M.M., Rosen D.S., Ornstein R.M., Mammel K.A., Katzman D.K., Rome E.S., Callahan S.T., Walsh B.T., Malizio J.B., Kearney S.A. (2013). Avoidant / restrictive food intake disorder: A proposed diagnosis in DSM-5 #journal#, 52(2 SUPPL. 1), S9, 0.922 | No data on untreated illness |
| 139 | Sileri P., Fabrizio I., Starr F., Franceschilli L., De Luca E., Di Giorgio A., Raniolo M., Bellini M.I., D'Ugo S., Gaspari A. (2012). Nervosa anorexia leads to defaecatory disorders compared to general population #journal#, 142(5 SUPPL. 1), S1072, 0.854 | Conference abstract |
| 140 | Via E., Soriano-Mas C., Sanchez I., Hernandez- Ribas R., Martinez-Zalacain I., Jimenez-Murcia S., Pujol J., Menchon J.M., Fernandez-Aranda F., Cardoner N. (2012). The effects of social judgment in anorexia nervosa: A functional magnetic resonance study #journal#, 22(SUPPL. 2), S198, 0.79 | No data on untreated illness |
| 141 | Runfola C., Payne A., Allison K., Lock J., Peebles R. (2012). Clinical severity of night eating syndrome in eating disordered adolescents: A case series #journal#, 50(2 SUPPL. 1), S62, 0.96 | No data on untreated illness |
| 142 | Treasure J. (2011). Eating disorders: State of the art #journal#, 26(SUPPL. 1), #Pages#, 0.884 | No data on untreated illness |
| 143 | Hergenroeder A., Dave A., Henges C. (2011). Outcome of patients with eating disorders treated at a large children's hospital #journal#, 48(2 SUPPL. 1), S50, 0.84 | No data on untreated illness |
| 144 | Jasik C., Breland D., Buckelew S. (2010). The association of pre-morbid obesity with severity of eating disorder presentation in males #journal#, 46(2 SUPPL. 1), S51, 0.869 | No data on untreated illness |
| 145 | Forman S., Grodin L., Rome E., Fisher M., Malizio J., Mammel K., Hergenroeder A., Buckelew S., Golden N., Woods E., Graham D., Rosen D., Kapphahn C., Callahan S.T., Sigel E., Bravender T., Peebles R., Romano M. (2010). An eleven site national quality improvement project of adolescent medicine-based eating disorders programs: Weight outcomes at one year #journal#, 46(2 SUPPL. 1), S9, 0.892 | No data on untreated illness |
| 146 | Merwin R.M., Zucker N.L., Lacy J.L., Elliott C.A. (2010). Interoceptive awareness in eating disorders: Distinguishing lack of clarity from non-acceptance of internal experience #journal#, 24(5), 892, 0.847 | No data on untreated illness |
| 147 | Gadas M., Skalska J., Remberk B., Namyslowska I., Siewierska A., Krempa-Kowalewska A. (2009). Cognitive impairments in adolescents with eating disorders and schizophrenia #journal#, 18(1), 59, 0.882 | No data on untreated illness |
| 148 | Perpina S.A., Lozano P. E., Navarro O.P., Miguel S.L., Garcia L.L., Castro-Fornieles J. (2008). Cognitive performance in adolescents with Anorexia Nervosa: Subgroups in relation to neuropsychological impairment #journal#, 18(S4), S283, 0.818 | No data on untreated illness |
| 149 | Strizzolo S., Favaro A., Tenconi E., Santonastaso P. (2004). Stressful life events and eating disorders #journal#, 10(1), 56, 0.841 | No data on untreated illness |
| 150 | Fernandez-Aranda F., Aitken A., Badia A., Gimenez L., Solano R., Collier D., Treasure J., Vallejo J. (2004). Personality and psychopathological traits of males with an eating disorder #journal#, 12(6), 367, 0.892 | No data on untreated illness |
| 151 | Kiriike N., Nagata T., Matsunaga H. (1996). Eating disorders and marriage #journal#, 36(2), 115, 0.926 | No data on untreated illness |
| 152 | Nunez C., Carbajal A., Moreiras O. (1994). Refeeding efficacy on body composition in hospitalized patients with anorexia nervosa #journal#, 14(10), 1457, 0.04 | No data on untreated illness |
| 153 | Stacher G., Bergmann H., Wiesnagrotzki S., Steiner-Mittelbach G., Kiss A., Abatzi T.-A. (1992). Primary anorexia nervosa: Gastric emptying and antral motor activity in 53 patients #journal#, 11(2), 163, 0.827 | No data on untreated illness |
| 154 | Le Grange D., Eisler I., Dare C., Russell G.F.M. (1992). Evaluation of family treatments in adolescent anorexia nervosa: A pilot study #journal#, 12(4), 347, 0.756 | No data on untreated illness |
| 155 | Kennedy S.H., Garfinkel P.E. (1989). Patients admitted to hospital with anorexia nervosa and bulimia nervosa: Psychopathology, weight gain, and attitudes toward treatment #journal#, 8(2), 181, 0.842 | No data on untreated illness |
| 156 | Stonehill E., Crisp A.H. (1977). Psychoneurotic characteristics of patients with anorexia nervosa before and after treatment and at follow up 4-7 years later #journal#, 21(3), 187, 0.828 | No data on untreated illness |
| 157 | Kurisu, Ken, Nohara, Nobuhiro, Inada, Shuji, Otani, Makoto, Noguchi, Haruko, Endo, Yuka, Sato, Yasuhiro, Fukudo, Shin, Nakazato, Michiko, Yamauchi, Tsuneo, Harada, Tomoko, Inoue, Koki, Hata, Tomokazu, Takakura, Shu, Sudo, Nobuyuki, Iida, Naoko, Mizuhara, Yuki, Wada, Yoshihisa, Ando, Tetsuya, Yoshiuchi, Kazuhiro (2023). Economic costs for outpatient treatment of eating disorders in Japan #journal#, 11(1), #Pages#, 0.09 | No data on untreated illness |
| 158 | Keegan, Ella, Waller, Glenn, Tchanturia, Kate, Wade, Tracey D. (2024). The potential value of brief waitlist interventions in enhancing treatment retention and outcomes: A randomised controlled trial #journal#, #volume#(#issue#), #Pages#, 0.083 | No data on untreated illness |
| 159 | Vollert, Bianka, Yim, See Heng, Görlich, Dennis, Beintner, Ina, Gordon, Gemma, Musiat, Peter, Schmidt, Ulrike, Jacobi, Corinna (2024). Using web-based, guided self-help to bridge the waiting time for face-to-face out-patient treatment for bulimic-spectrum disorders: Randomised controlled trial #journal#, 10(#issue#), #Pages#, 0.078 | No data on untreated illness |
| 160 | Forrest, Lauren N., Grilo, Carlos M. (2022). Change in eating-disorder psychopathology network structure in patients with binge-eating disorder: Findings from treatment trial with 12-month follow-up #journal#, 90(6), 491, 0.084 | No data on untreated illness |
| 161 | Lammers, Mirjam W., Vroling, Maartje S., Crosby, Ross D., van Strien, Tatjana (2022). Dialectical behavior therapy compared to cognitive behavior therapy in binge‐eating disorder: An effectiveness study with 6‐month follow‐up #journal#, 55(7), 902, 0.082 | No data on untreated illness |
| 162 | Romano, Kelly A., Lipson, Sarah K., Beccia, Ariel L., Quatromoni, Paula A., Murgueitio, Jose (2023). Disparities in eating disorder symptoms and mental healthcare engagement prior to and following the onset of the COVID‐19 pandemic: Findings from a national study of US college students #journal#, 56(1), 203, 0.871 | No data on untreated illness |
| 163 | Levallius, Johanna (2022). Eating disorders and personality #journal#, 83(2-B), #Pages#, 0.797 | Inappropriate study design or population |
| 164 | Riquin, Elise, Raynal, Agathe, Mattar, Lama, Lalanne, Christophe, Hirot, France, Huas, Caroline, Duclos, Jeanne, Berthoz, Sylvie, Godart, Nathalie (2021). 'Is the severity of the clinical expression of anorexia nervosa influenced by an anxiety, depressive, or obsessive-compulsive comorbidity over a lifetime': Erratum #journal#, 12(#issue#), #Pages#, 0.847 | Inappropriate study design or population |
| 165 | Coelho, Jennifer S., Suen, Janet, Marshall, Sheila, Burns, Alex, Lam, Pei-Yoong, Geller, Josie (2021). Parental experiences with their child’s eating disorder treatment journey #journal#, 9(#issue#), #Pages#, 0.686 | No data on untreated illness |
| 166 | Trottier, Kathryn, Monson, Candice M., Wonderlich, Stephen A., Crosby, Ross D. (2022). Results of the first randomized controlled trial of integrated cognitive-behavioral therapy for eating disorders and posttraumatic stress disorder #journal#, 52(3), 587, 0.917 | No data on untreated illness |
| 167 | Trottier, Kathryn, Monson, Candice M., Wonderlich, Stephen A., Crosby, Ross D. (2022). 'Results of the first randomized controlled trial of integrated cognitive-behavioral therapy for eating disorders and posttraumatic stress disorder': Corrigendum #journal#, 52(3), 600, 0.892 | No data on untreated illness |
| 168 | Anderson, Lisa M., Smith, Kathryn E., Nuñez, Mia C., Farrell, Nicholas R. (2021). Family accommodation in eating disorders: A preliminary examination of correlates with familial burden and cognitive-behavioral treatment outcome #journal#, 29(4), 327, 0.036 | No data on untreated illness |
| 169 | Guinhut, Marie, Godart, Nathalie, Benadjaoud, Mohamed-Amine, Melchior, Jean-Claude, Hanachi, Mouna (2021). Five‐year mortality of severely malnourished patients with chronic anorexia nervosa admitted to a medical unit #journal#, 143(2), 130, 0.023 | No data on untreated illness |
| 170 | Dingemans, Alexandra E., van Son, Gabriëlle E., Vanhaelen, Christine B., Furth, Eric F. (2020). Depressive symptoms rather than executive functioning predict group cognitive behavioural therapy outcome in binge eating disorder #journal#, 28(6), 620, 0.838 | No data on untreated illness |
| 171 | Støving, René Klinkby, Larsen, Pia Veldt, Winkler, Laura Al‐Dakhiel, Bilenberg, Niels, Røder, Michael Ejnar, Steinhausen, Hans‐Christoph (2020). Time trends in treatment modes of anorexia nervosa in a nationwide cohort with free and equal access to treatment #journal#, 53(12), 1952, 0.01 | No data on untreated illness |
| 172 | Riedlinger, Caroline, Schmidt, Greta, Weiland, Alisa, Stengel, Andreas, Giel, Katrin Elisabeth, Zipfel, Stephan, Enck, Paul, Mack, Isabelle (2020). Which symptoms, complaints and complications of the Gastrointestinal tract occur in patients with eating disorders? A systematic review and quantitative analysis #journal#, 11(#issue#), #Pages#, 0.896 | No data on untreated illness |
| 173 | Seriès, Claire, Mainhagu, Pierre, de Boucaud, Michel (2017). Anorexie mentale masculine = Anorexia nervosa in men #journal#, 175(10), 918, 0.016 | No data on untreated illness |
| 174 | Mingione, Carolyn J. (2018). Psychometric properties of the Yale-Brown Obsessive Compulsive Scale modified for Binge Eating in individuals with binge eating disorder #journal#, 79(7-B(E)), #Pages#, 0.039 | No data on untreated illness |
| 175 | Murray, Stuart B., Loeb, Katharine L., Le Grange, Daniel (2018). Treatment outcome reporting in anorexia nervosa: Time for a paradigm shift? #journal#, 6(#issue#), #Pages#, 0.004 | No data on untreated illness |
| 176 | Bowen, Megan M., Lambert, Michael J., Berkeljon, Arjan, Orr, Tyler E., Berrett, Michael, Simon, Witold (2016). Effects of feedback-assisted treatment on post-treatment outcome for eating disordered inpatients: A follow-up study #journal#, 3(1), #Pages#, 0.003 | No data on untreated illness |
| 177 | Folke, Sofie, Daniel, Sarah I. F., Gondan, Matthias, Lunn, Susanne, Tækker, Louise, Poulsen, Stig (2017). Therapist adherence is associated with outcome in cognitive–behavioral therapy for bulimia nervosa #journal#, 54(2), 195, 0.007 | No data on untreated illness |
| 178 | Bowen, Megan Michelle (2016). Effects of feedback assisted treatment on post-treatment outcome for eating disordered inpatients: A follow-up study #journal#, 77(3-B(E)), #Pages#, 0.016 | No data on untreated illness |
| 179 | Bourdier, Léna, Maria, Anne-Solène, Ringuenet, Damien, Berthoz, Sylvie (2016). Intelligence émotionnelle et anorexie mentale: Liens avec la symptomatologie alimentaire et perspectives thérapeutiques = Emotional intelligence and anorexia nervosa: Links with eating disorders symptoms and therapeutic perspectives #journal#, 26(1), 12, 0.072 | No data on untreated illness |
| 180 | Leslie, Jennifer (2016). Understanding the changing landscape of client perspectives of recovery from Anorexia Nervosa #journal#, 76(11-B(E)), #Pages#, 0.009 | Inappropriate study design or population |
| 181 | Frank, Guido K. W. (2015). What causes eating disorders, and what do they cause? #journal#, 77(7), 602, 0.022 | No data on untreated illness |
| 182 | Schmidt, Ulrike, Brown, Amy, McClelland, Jessica, Glennon, Danielle, Mountford, Victoria A. (2016). Will a comprehensive, person-centered, team‐based early intervention approach to first episode illness improve outcomes in eating disorders? #journal#, 49(4), 374, 0.966 | No data on untreated illness |
| 183 | Fonville, L., Giampietro, V., Williams, S. C. R., Simmons, A., Tchanturia, K. (2014). 'Alterations in brain structure in adults with anorexia nervosa and the impact of illness duration': Corrigendum #journal#, 44(9), 1976, 0.096 | No data on untreated illness |
| 184 | Darcy, Alison M., Fitzpatrick, Kathleen K., Lock, James (2015). Cognitive remediation therapy and cognitive-behavioral therapy for anorexia nervosa #journal#, #volume#(#issue#), 102, 0.027 | No data on untreated illness |
| 185 | Castellini, Giovanni, Montanelli, Luca, Faravelli, Carlo, Ricca, Valdo (2014). Eating disorder outpatients who do not respond to cognitive behavioral therapy: A follow-up study #journal#, 83(2), 125, 0.816 | Inappropriate study design or population |
| 186 | Ivanova, Iryna (2015). The 'how' of change in emotion-focused group therapy for eating disorders #journal#, 76(4-B(E)), #Pages#, 0.079 | No data on untreated illness |
| 187 | Daniel, Sarah I. F., Lunn, Susanne, Poulsen, Stig (2014). Autobiographical memory narratives in treatment for bulimia nervosa: The effect of client attachment, depression, and therapy type #journal#, 24(1), 153, 0.008 | No data on untreated illness |
| 188 | Crow, Scott (2013). Eating disorders and risk of death #journal#, 170(8), 824, 0.021 | Inappropriate study design or population |
| 189 | Sim, Leslie A., Lebow, Jocelyn, Billings, Marcie (2013). Eating disorders in adolescents with a history of obesity #journal#, 132(4), e1026, 0.862 | No data on untreated illness |
| 190 | Higgins, Janine, Hagman, Jennifer, Pan, Zhaoxing, MacLean, Paul (2013). Increased physical activity not decreased energy intake is associated with inpatient medical treatment for anorexia nervosa in adolescent females #journal#, 8(4), #Pages#, 0.017 | No data on untreated illness |
| 191 | Dennis, Amy Baker, Pryor, Tamara (2014). Introduction to eating disorders for substance abuse specialists #journal#, #volume#(#issue#), 199, 0.055 | No data on untreated illness |
| 192 | Pérez-Pareja, Brígida, Quiles, Yolanda, Romero, Cristina, Pamies-Aubalat, Lidia, Quiles, María J. (2014). Malestar psicológico y emoción expresada en cuidadores de pacientes con un trastorno de la conducta alimentaria = Expressed emotion and psychological distress in caregivers of patients with an eating disorder #journal#, 30(1), 37, 0.114 | No data on untreated illness |
| 193 | Karlsson, Gunilla Paulson, Clinton, David, Nevonen, Lauri (2013). Prediction of weight increase in anorexia nervosa #journal#, 67(6), 424, 0.068 | No data on untreated illness |
| 194 | Abbate-Daga, Giovanni, Amianto, Federico, Delsedime, Nadia, De-Bacco, Carlotta, Fassino, Secondo (2013). Resistance to treatment and change in anorexia nervosa: A clinical overview #journal#, 13(#issue#), #Pages#, 0.032 | No data on untreated illness |
| 195 | Calderoni, Sara, Fantozzi, Pamela, Maestro, Sandra, Narzisi, Antonio, Muratori, Filippo (2012). Deficit di empatia cognitiva in adolescenti con anoressia nervosa di tipo restrittivo = Impaired cognitive empathy in adolescents with restrictive type anorexia nervosa #journal#, 11(2), 81, 0.919 | No data on untreated illness |
| 196 | Greif, Rebecca Anya (2013). Reducing eating disorder risk factors in members of a national sorority: A benchmarking study #journal#, 74(4-B(E)), #Pages#, 0.148 | No data on untreated illness |
| 197 | Waller, Glenn, Evans, Jane, Stringer, Hannah (2012). The therapeutic alliance in the early part of cognitive‐behavioral therapy for the eating disorders #journal#, 45(1), 63, 0.055 | No data on untreated illness |
| 198 | Kafantaris, Vivian, Leigh, Ellen, Hertz, Stanley, Berest, Alison, Schebendach, Janet, Sterling, Wendy Meyer, Saito, Ema, Sunday, Suzanne, Higdon, Claudine, Golden, Neville H., Malhotra, Anil K. (2011). A placebo-controlled pilot study of adjunctive olanzapine for adolescents with anorexia nervosa #journal#, 21(3), 207, 0.047 | No data on untreated illness |
| 199 | Zalar, Bojan, Weber, Urška, Sernec, Karin (2011). Aggression and impulsivity with impulsive behaviours in patients with purgative anorexia and bulimia nervosa #journal#, 23(1), 27, 0.075 | No data on untreated illness |
| 200 | Vaz-Leal, Francisco J., Santos, Laura Rodríguez, García-Herráiz, M. Angustias, Monge-Bautista, Melchora, López-Vinuesa, Beatriz (2011). Bulimia nervosa with history of anorexia nervosa: Could the clinical subtype of anorexia have implications for clinical status and treatment response? #journal#, 44(3), 212, 0.06 | Inappropriate study design or population |
| 201 | Zucker, Nancy, Moskvich, Ashley, Soo, Adrienne (2011). Neuropsychological aspects of eating disorders #journal#, 41(11), 539, 0.11 | No data on untreated illness |
| 202 | Runfola, Cristin D. (2012). Night eating syndrome: Prevalence and clinical correlates in pediatric eating disorder patients #journal#, 73(2-B), 1265, 0.896 | Inappropriate study design or population |
| 203 | Hartmann, Armin, Weber, Stefanie, Herpertz, Stephan, Zeeck, Almut (2011). Psychological treatment for anorexia nervosa: A meta-analysis of standardized mean change #journal#, 80(4), 216, 0.004 | No data on untreated illness |
| 204 | Kovács (Krizbai), Tímea, Szabó, Pál, Pászthy, Bea (2011). Reduced specificity of autobiographical memory in anorexia nervosa #journal#, 11(1), 57, 0.082 | No data on untreated illness |
| 205 | Wilson, G. Terence (2011). Treatment of binge eating disorder #journal#, 34(4), 773, 0.049 | No data on untreated illness |
| 206 | Waugh, Esther J. (2010). An investigation of the associations among recovery, key illness characteristics and bone mineral density in women with a history of anorexia nervosa #journal#, 71(6-B), 3610, 0.019 | No data on untreated illness |
| 207 | Hill, Diana Marie (2009). Appetite-focused dialectical behavior therapy for the treatment of binge eating with purging: A randomized controlled trial #journal#, 69(7-B), 4424, 0.017 | No data on untreated illness |
| 208 | Waller, Glenn, Schmidt, Ulrike, Treasure, Janet, Emanuelli, Francesca, Alenya, Joana, Crockett, Jo, Murray, Katie (2009). Ethnic origins of patients attending specialist eating disorders services in a multiethnic urban catchment area in the United Kingdom #journal#, 42(5), 459, 0.067 | No data on untreated illness |
| 209 | Couturier, Jennifer, Isserlin, Leanna, Lock, James (2010). Family-based treatment for adolescents with anorexia nervosa: A dissemination study #journal#, 18(3), 199, 0.013 | Inappropriate study design or population |
| 210 | Cullum, Jodi L. (2009). Maladaptive schemas as a predictor of residential treatment outcomes in females with eating disorders #journal#, 70(11-B), 7227, 0.029 | No data on untreated illness |
| 211 | Domiano, Pietro, Sironi, Rossella, Gibin, Anna Maria, Miglioli, Maristella, Brunori, Luisa (2010). Psicoterapia di gruppo per i DCA: Studio sull'efficacia = Study on the effectiveness of group psychotherapy for DCA patients #journal#, 12(2), 69, 0.843 | No data on untreated illness |
| 212 | García, Anna Carretero, Planell, Luís Sánchez, Estragués, Jordi Rusiñol, Escursell, Rosa M. Raich, Carracedo, David Sánchez (2010). Relevant risk factors, current eating psychopathology, body shape concern and psychological functioning in eating disorders #journal#, 14(1), 32, 0.872 | No data on untreated illness |
| 213 | Downe, Kristina A., Goldfein, Juli A., Devlin, Michael J. (2009). Restraint, hunger, and disinhibition following treatment for binge-eating disorder #journal#, 42(6), 498, 0.012 | No data on untreated illness |
| 214 | Kaye, Walter, Bailer, Ursula, Frank, Guido, Henry, Shannan, Price, Julie, Meltzer, Carolyn, Becker, Carl, Ziolko, Scott, Mathis, Chester, Wagner, Angela, Barbarich-Marsteller, Nicole, Putnam, Karen (2008). Serotonin transporter binding after recovery from eating disorders #journal#, 197(3), 521, 0.011 | Inappropriate study design or population |
| 215 | Pereira, Tintina (2010). The therapeutic alliance and outcome in anorexia nervosa #journal#, 70(8-B), 5180, 0.021 | No data on untreated illness |
| 216 | Edwards, Christopher L., Rickel, Katie (2008). An introduction to eating disorder and weight management #journal#, 53(26), #Pages#, 0.032 | No data on untreated illness |
| 217 | Reiff, Dan W. (2007). Attentional biases in women with anorexia nervosa and those who have recovered #journal#, 67(9-B), 5437, 0.037 | No data on untreated illness |
| 218 | Vollmann, Jochen (2006). 'But I don't feel it': Values and emotions in the assessment of competence in patients with anorexia nervosa #journal#, 13(4), 289, 0.009 | No data on untreated illness |
| 219 | Latzer, Yael, Hochdorf, Zipora (2005). Dying to live: Attachment to life in Anorexia Nervosa #journal#, #volume#(#issue#), 157, 0.019 | No data on untreated illness |
| 220 | Carney, Terry, Ingvarson, Mim, Tait, David (2006). Experiences of 'control' in anorexia nervosa treatment: Delayed coercion, shadow of law, or disseminated power and control? #journal#, #volume#(#issue#), 41, 0.012 | No data on untreated illness |
| 221 | Le Grange, Daniel (2004). Family-based treatment for adolescent anorexia nervosa: A promising approach? #journal#, 8(2), 56, 0.013 | No data on untreated illness |
| 222 | Tantleff-Dunn, Stacey, Gokee-LaRose, Jessica, Peterson, Rachel D. (2004). Interpersonal Psychotherapy for the Treatment of Anorexia Nervosa, Bulimia Nervosa, and Binge Eating Disorder #journal#, #volume#(#issue#), 163, 0.048 | No data on untreated illness |
| 223 | Cockell, Sarah J., Zaitsoff, Shannon L., Geller, Josie (2004). Maintaining change following eating disorder treatment #journal#, 35(5), 527, 0.072 | No data on untreated illness |
| 224 | Krautter, Tonja Heidi (2002). A descriptive study of the perceived reasons for the effectiveness of a time-limited, family-based therapy approach for adolescents diagnosed with anorexia nervosa #journal#, 63(6-B), 3011, 0.014 | No data on untreated illness |
| 225 | Tanaka, Muneki, Naruo, Tetsuro, Nagai, Nobuatsu, Kuroki, Nobutaka, Shiiya, Tomomi, Nakazato, Masamitsu, Matsukura, Shigeru, Nozoe, Shin-ichi (2003). Corrigendum to 'Habitual binge/purge behaviour influences circulating ghrelin levels in eating disorders' #journal#, 37(3), 265, 0.036 | No data on untreated illness |
| 226 | Campbell, Laura Anne (2003). Dimensions of perfectionism in eating disorders: Diagnostic specificity and prognostic significance #journal#, 64(6-B), 2909, 0.923 | No data on untreated illness |
| 227 | Mahon, Jennifer, Winston, Anthony P., Palmer, Robert L., Harvey, Peter K. (2001). Do broken relationships in childhood relate to bulimic women breaking off psychotherapy in adulthood? #journal#, 29(2), 139, 0.06 | No data on untreated illness |
| 228 | Thompson-Brenner, Heather Jean (2003). Implications for the treatment of bulimia nervosa: A meta-analysis of efficacy trials and a naturalistic study of treatment in the community #journal#, 63(10-B), 4928, 0.021 | No data on untreated illness |
| 229 | Babiss, Fran (2001). Mental health treatment and its aftermath: The experiences of three women diagnosed with mental illness #journal#, 61(10-B), 5550, 0.008 | No data on untreated illness |
| 230 | Stewart, Anne (2004). Prevention of Eating Disorders #journal#, #volume#(#issue#), 173, 0.058 | No data on untreated illness |
| 231 | Carter, Frances A., McIntosh, Virginia V. W., Joyce, Peter R., Sullivan, Patrick F., Bulik, Cynthia M. (2003). Role of exposure with response prevention in cognitive-behavioral therapy for bulimia nervosa: Three-year follow-up results #journal#, 33(2), 127, 0.014 | No data on untreated illness |
| 232 | Stickney, Marcella I., Miltenberger, Raymond G., Wolff, Gretchen (1999). A descriptive analysis of factors contributing to binge eating #journal#, 30(3), 177, 0.012 | No data on untreated illness |
| 233 | Rossotto, Elizabeth (1998). Bulimia nervosa with and without substance use disorders: A comparative study #journal#, 58(8-B), 4469, 0.1 | No data on untreated illness |
| 234 | Noordenbos, Greta, Jacobs, Mariel E., Hertzberger, Elize (1998). Chronic eating disorders: The patients' view of their treatment history #journal#, 6(3), 217, 0.041 | Inappropriate study design or population |
| 235 | Crawford, Angela Lynn (1998). Environmental contexts and functional impairment associated with help-seeking and behavior change among women with bulimia #journal#, 58(10-B), 5639, 0.041 | No data on untreated illness |
| 236 | Hudson, James I., Chase, Emily A., Pope, Harrison G. Jr. (1998). Eye movement desensitization and reprocessing in eating disorders: Caution against premature acceptance #journal#, 23(1), 1, 0.02 | No data on untreated illness |
| 237 | Birchall, Helen (1999). Interpersonal psychotherapy in the treatment of eating disorders #journal#, 7(5), 315, 0.054 | No data on untreated illness |
| 238 | McElroy, Susan L., Casuto, Leah S., Nelson, Eric B., Lake, Kathleen A., Soutullo, Cesar A., Keck, Paul E. Jr., Hudson, James I. (2000). Placebo-controlled trial of sertraline in the treatment of binge eating disorder #journal#, 157(6), 1004, 0.077 | No data on untreated illness |
| 239 | Tappe, Karyn A., Gerberg, Susan E., Shide, David J., Rolls, Barbara J., Andersen, Arnold A. (1998). Videotape assessment of changes in aberrant meal-time behaviors in anorexia nervosa after treatment #journal#, 30(2), 171, 0.027 | No data on untreated illness |
| 240 | Shure, Jane Karen (1996). A needs assessment toward developing a model training agenda for the brief treatment of eating disorders #journal#, 57(3-B), 1696, 0.016 | No data on untreated illness |
| 241 | Gleaves, David H., Post, Gail K., Eberenz, Kathleen P., Davis, William N. (1993). A report of 497 women hospitalized for treatment of bulimia nervosa #journal#, 1(2), 134, 0.114 | No data on untreated illness |
| 242 | Samuel-Lajeunesse, B., Criquillon-Doublet, S., Divac, S., Romo Jiménez, M. L. (1995). Aspectos cualitativos y modalidades evolutivas de los trastornos de conducta alimenticia = Qualitative aspects and evolutional modalities of eating disorders #journal#, 11(3), 103, 0.027 | No data on untreated illness |
| 243 | Jones, Rosemary, Peveler, Robert C., Hope, R. A., Fairburn, Christopher G. (1993). Changes during treatment for bulimia nervosa: A comparison of three psychological treatments #journal#, 31(5), 479, 0.021 | Inappropriate study design or population |
| 244 | Galante, Danielle Marie (1995). Characteristics of families with anorexic or bulimic daughters: A study of treatment phases #journal#, 55(8-A), 2589, 0.057 | No data on untreated illness |
| 245 | Mickley, Diane W. (1994). Medical aspects of anorexia and bulimia #journal#, #volume#(#issue#), 7, 0.003 | No data on untreated illness |
| 246 | Pryor, Tamara L., Johnson, Theresa, Wiederman, Michael W., Boswell, Donald L. (1995). The clinical significance of symptom denial among women with anorexia nervosa: Another disposable myth? #journal#, 3(4), 293, 0.025 | No data on untreated illness |
| 247 | Anderson, Nicolle Woodbury (1996). The relationship between sex role conflict and eating disorders in women #journal#, 57(6-B), 4021, 0.083 | No data on untreated illness |
| 248 | Griffiths, Rosalyn A., Hadzi-Pavlovic, Dusan, Channon-Little, Lorna (1996). The short-term follow-up effects of hypnobehavioural and cognitive behavioural treatment for bulimia nervosa #journal#, 4(1), 12, 0.082 | No data on untreated illness |
| 249 | Lowenkopf, Eugene L., Wallach, Judith D. (1985). Bulimia: Theoretical conceptualizations and therapies #journal#, 13(4), 489, 0.038 | No data on untreated illness |
| 250 | Tobin, David L., Johnson, Craig L., Franke, Kevin (1992). Clinical treatment of eating disorders #journal#, #volume#(#issue#), 330, 0.014 | No data on untreated illness |
| 251 | Witcher, Darlene B., Williamson, Donald A. (1992). Duration of bulimia nervosa and symptom progression: A retrospective analysis of treatment-seeking bulimics #journal#, 4(3), 255, 0.169 | No data on untreated illness |
| 252 | Brotman, Andrew W., Alonso, Anne, Herzog, David B. (1985). Group therapy for bulimia: Clinical experience and practical recommendations #journal#, 9(1), 15, 0.022 | No data on untreated illness |
| 253 | Riess, Helen, Rutan, J. Scott (1992). Group therapy for eating disorders: A step-wise approach #journal#, 16(2), 79, 0.036 | No data on untreated illness |
| 254 | Paul, Thomas, Weitemeyer, Erika, Pudel, Volker (1986). Telefonische Beratung bei Bulimia nervosa = Telephone consultations in bulimia nervosa #journal#, 34(4), 315, 0.047 | No data on untreated illness |
| 255 | Brisman, Judith, Siegel, Michele (1985). The bulimia workshop: A unique integration of group treatment approaches #journal#, 35(4), 585, 0.033 | No data on untreated illness |
| 256 | Mitchell, James E., Soll, Elizabeth, Eckert, Elke O., Pyle, Richard L., Hatsukami, D. (1989). The changing population of bulimia nervosa patients in an eating disorders program #journal#, 40(11), 1188, 0.092 | No data on untreated illness |
| 257 | Robinson, M., Puckett, S., McNeely, M., Lunsford, J. (2023). Disease Activity and Duration Since Diagnosis Do Not Affect Food Neophobia in IBD...Food and Nutrition Conference and Expo, October 7-10, 2023, Denver, Colorado. #journal#, 123(10), A31, 0.069 | No data on untreated illness |
| 258 | Ranzenhofer, Lisa M., Jablonski, Monica, Davis, Lauren, Posner, Jonathan, Walsh, B. Timothy, Steinglass, Joanna E. (2022). Early Course of Symptom Development in Anorexia Nervosa. #journal#, 71(5), 587, 0.84 | No data on untreated illness |
| 259 | Babb, Charli, Brede, Janina, Jones, Catherine R. G., Serpell, Lucy, Mandy, William, Fox, John (2022). A comparison of the eating disorder service experiences of autistic and non‐autistic women in the UK. #journal#, 30(5), 616, 0.912 | No data on untreated illness |
| 260 | Craig, Mirin, Waine, Joanne, Wilson, Scott, Waller, Glenn (2019). Optimizing treatment outcomes in adolescents with eating disorders: The potential role of cognitive behavioral therapy. #journal#, 52(5), 538, 0.938 | No data on untreated illness |
| 261 | Wagner, Gudrun, Zeiler, Michael, Berger, Gabriele, Huber, Wolf-Dietrich, Favaro, Angela, Santonastaso, Paolo, Karwautz, Andreas (2015). Eating Disorders in Adolescents with Celiac Disease: Influence of Personality Characteristics and Coping. #journal#, 23(5), 361, 0.933 | No data on untreated illness |
| 262 | Adambegan, Mandana, Wagner, Gudrun, Nader, Ingo W., Fernandez-Aranda, Fernando, Treasure, Janet, Karwautz, Andreas (2012). Internalizing and Externalizing Behaviour Problems in Childhood Contribute to the Development of Anorexia and Bulimia Nervosa-A Study Comparing Sister Pairs. #journal#, 20(2), 116, 0.956 | No data on untreated illness |
| 263 | Eisenberg, Daniel, Nicklett, Emily J., Roeder, Kathryn, Kirz, Nina E. (2011). Eating Disorder Symptoms Among College Students: Prevalence, Persistence, Correlates, and Treatment-Seeking. #journal#, 59(8), 700, 0.848 | Inappropriate study design or population |
| 264 | Currin L, Schmidt U (2005). A critical analysis of the utility of an early intervention approach in the eating disorders. #journal#, 14(6), 611, 0.844 | No data on untreated illness |
| 265 | Milos G, Spindler A, Ruggiero G, Klaghofer R, Schnyder U (2002). Comorbidity of obsessive-compulsive disorders and duration of eating disorders. #journal#, 31(3), 284, 0.933 | No data on untreated illness |
| 266 | Herpertz S, Albus C, Lichtblau K, Köhle K, Mann K, Senf W (2000). Relationship of weight and eating disorders in type 2 diabetic patients: a multicenter study. #journal#, 28(1), 68, 0.962 | No data on untreated illness |
| 267 | Peeters F, Meijboom A (2000). Electrolyte and other blood serum abnormalities in normal weight bulimia nervosa: evidence for sampling bias. #journal#, 27(3), 358, 0.844 | No data on untreated illness |
| 268 | Goodrick GK, Pendleton VR, Kimball KT, Poston WSC, Reeves RS, Foreyt JP (1999). Binge eating severity, self-concept, dieting self-efficacy and social support during treatment of binge eating disorder. #journal#, 26(3), 295, 0.812 | No data on untreated illness |
| 269 | Strouphauer, E., Suhail, S., Mulinda, C., Ufomadu, P., Nyamongo, N., Lee, G., & Kim, S. J. (2024). Prevalence of psychiatric comorbidities and treatment initiation in African American pediatric patients with vitiligo: A retrospective, single-center, case-control study. JAAD international, 17, 104–110. | No data on untreated illness |
| 270 | Grycuk, L., Monssen, D., Davies, M. R., Sharpe, H., Allen, K. L., Prasad, V., Potterton, R., Popat, P., Mountford, V. A., McNeil, S., Lawrence, V., Grant, N., Goldsmith, K. A., Glennon, D., Byford, S., Brown, A., & Schmidt, U. (2025). Shortening duration of untreated illness in young people with first episode eating disorders: protocol of a randomised controlled feasibility trial of a smartphone friendly multi-modal decision-making tool (FREED-M) to improve help-seeking. Pilot and feasibility studies, 11(1), 14. | Study protocol |
| 271 | Preti, A., Bevione, F., Lacidogna, M. C., Raballo, A., Poletti, M., & Abbate-Daga, G. (2025). Early intervention in eating disorders: introducing the chronopathogram. Eating and weight disorders : EWD, 30(1), 9. | Inappropriate study design or population |
| 272 | Munguía, L., Baenas, I., Granero, R., Ohsako, N., Gaspar-Pérez, A., Perales, I., Rosinska, M., Sánchez-Díaz, I., Toro, J. J., Sánchez-González, J., Arcelus, J., Paslakis, G., Jiménez-Murcia, S., & Fernández-Aranda, F. (2025). Eating disorder debut cases during COVID-19 lockdown in adults. Exploring differences in treatment outcome contrasting with pre-pandemic onset cases. European eating disorders review : the journal of the Eating Disorders Association, 33(2), 304–317. | No data on untreated illness |
| 273 | Mannan, H., Palavras, M. A., Claudino, A., & Hay, P. J. (2025). Predictors and Moderators of Outcomes in a Trial of Cognitive Behavioural Therapy Integrated with Behavioural Weight Loss for High Weight Individuals with Disorders of Recurrent Binge Eating. Nutrients, 17(7), 1288. | No data on untreated illness |
| 274 | Hercus, C., Baird, A., Ibrahim, S., Turnbull, P., Appleby, L., Singh, U., & Kapur, N. (2024). Suicide in individuals with eating disorders who had sought mental health treatment in England: a national retrospective cohort study. The lancet. Psychiatry, 11(8), 592–600. | No data on untreated illness |
| 275 | Käver, L., Voelz, C., Specht, H. E., Thelen, A. C., Keller, L., Dahmen, B., Andreani, N. A., Tenbrock, K., Biemann, R., Borucki, K., Dempfle, A., Baines, J. F., Beyer, C., Herpertz-Dahlmann, B., Trinh, S., & Seitz, J. (2024). Cytokine and Microbiome Changes in Adolescents with Anorexia Nervosa at Admission, Discharge, and One-Year Follow-Up. Nutrients, 16(11), 1596. | No data on untreated illness |
| 276 | Liao, Z., Birgegård, A., Monell, E., Borg, S., Bulik, C. M., & Mantilla, E. F. (2024). Maladaptive exercise in eating disorders: lifetime and current impact on mental health and treatment seeking. Journal of eating disorders, 12(1), 86. | No data on untreated illness |
| 277 | Hansen, N. K., Ries, E. S., Ward, T., Cardi, V., Christensen, A. B., Hjorthøj, C., Nordentoft, M., Micali, N., & Glenthøj, L. B. (2025). The Dialogue study: Protocol for a randomized clinical trial evaluating the efficacy of virtual reality-based psychotherapy plus treatment as usual versus treatment as usual for eating disorders. PloS one, 20(7), e0319875. | Preprint |
| 278 | Larsen, J. T., Chatwin, H., Zhang, Z. P., Clausen, L., Bulik, C. M., Thornton, L. M., Micali, N., Petersen, L. V., & Yilmaz, Z. (2025). Construction of a register-based severity index for anorexia nervosa in Denmark: Association with overall and cause-specific mortality. European psychiatry : the journal of the Association of European Psychiatrists, 68(1), e104. | Preprint |
| 279 | Newberry-Dupe, J., Melvin, G., King, K., White, M. J., Anderson, V., Babl, F. E., Borland, M. L., Dalziel, S. R., Hiscock, H., Tonge, B. J., Buntine, P., Charters, B., Hamilton, M., Kochar, A., Meyer, A., Tran, V., Aldridge, E., Barrington, G., Hackett, L., Lowry, N., … Craig, S. (2025). Paediatric Emergency Department Mental Health and Behavioural Presentations in Australia Before and After the Onset of the COVID-19 Pandemic: Retrospective Observational Study. Journal of paediatrics and child health, 61(6), 910–918. | No data on untreated illness |
| 280 | Biancalani, A., Occhionero, M., Leuci, E., Quattrone, E., Azzali, S., Paulillo, G., Pupo, S., Pellegrini, P., Menchetti, M., & Pelizza, L. (2025). Disorganization in individuals at clinical high risk for psychosis: psychopathology and treatment response. European archives of psychiatry and clinical neuroscience, 275(3), 921–935. | No data on untreated illness |
| 281 | Shepherd, Caitlin et al. Journal of Adolescent Health, Volume 76, Issue 3, S26. Preliminary Impact of Illness Duration on Psychological Outcomes for Adolescents With Anorexia Nervosa Diagnoses in an Enhanced Remote Partial Hospitalization/Intensive Outpatient Program for Eating Disorders | Conference abstract |
| 282 | Downey, Amanda et al., Journal of Adolescent Health, Volume 76, Issue 3, S29. Hypometabolism in Patients With Atypical Anorexia Nervosa: Baseline Findings From the Individualized-Study of Refeeding to Optimize Inpatient Gains (Istrong) | Conference abstract |
| 283 | Calugi, S., Dalle Grave, A., Chimini, M., Lorusso, A., & Dalle Grave, R. (2024). Illness duration and treatment outcome of intensive cognitive-behavioral therapy in adolescents with anorexia nervosa. The International journal of eating disorders, 57(7), 1566–1575. | No data on untreated illness |
| 284 | Breiner, C. E., Knedgen, M. M., Proctor, K. B., & Zickgraf, H. F. (2024). Relation between ARFID symptomatology and picky eating onset and duration. Eating behaviors, 54, 101900. | No data on untreated illness |
| 285 | Hebebrand, J., Gradl-Dietsch, G., Peters, T., Correll, C. U., & Haas, V. (2024). The Diagnosis and Treatment of Anorexia Nervosa in Childhood and Adolescence. Deutsches Arzteblatt international, 121(5), 164–174. | Inappropriate study design or population |
| 286 | Eimear Doody, Karen M. Ryan, Clare O'Toole, Declan M. McLoughlin (2024), Duration of anorexia nervosa is positively associated with whole blood mitochondrial DNA copy number: A cross-sectional case-control study, The European Journal of Psychiatry, Volume 38, Issue 4. | No data on untreated illness |
| 287 | Manwaring, J. L., Blalock, D. V., Duffy, A., Le Grange, D., Mehler, P. S., Riddle, M., & Rienecke, R. D. (2024). An examination of adults with atypical anorexia nervosa at admission to treatment at higher levels of care: An attempt to increase diagnostic clarity. The International journal of eating disorders, 57(4), 848–858. | Inappropriate study design or population |
| 288 | Lopes, M. P., Ahmed, S., Beaman, L., Stubbs, B., Campbell, I. C., Schmidt, U., & Robinson, L. (2025). Bone Fracture History in Women With First Episode or With Persistent Anorexia Nervosa. European eating disorders review : the journal of the Eating Disorders Association, 33(3), 447–459. | No data on untreated illness |
| 289 | Baker, J. H., Freestone, D., Cai, K., Silverstein, S., Urban, B., & Steinberg, D. (2024). Eating Disorder Clinical Presentation and Treatment Outcomes by Gender Identity Among Children, Adolescents, and Young Adults. The Journal of adolescent health : official publication of the Society for Adolescent Medicine, 75(2), 254–260. | No data on untreated illness |
| 290 | Slof-Op 't Landt, M. C. T., Dingemans, A. E., & Giltay, E. J. (2022). Eating disorder psychopathology dimensions based on individual co-occurrence patterns of symptoms over time: a dynamic time warp analysis in a large naturalistic patient cohort. Eating and weight disorders : EWD, 27(8), 3649–3663. | No data on untreated illness |
| 291 | Vanzhula, I., Hagan, K., Duck, S. A., Pan, I., Wang, E. Y., Steinglass, J., Attia, E., Wildes, J. E., Guarda, A. S., & Schreyer, C. (2024). Eating disorder symptom non-endorsers in hospitalised patients with anorexia nervosa: Who are they?. European eating disorders review : the journal of the Eating Disorders Association, 32(4), 795–808. | No data on untreated illness |
| 292 | Riedlinger, C., Mazurak, N., Schäffeler, N., Stengel, A., Giel, K. E., Zipfel, S., Enck, P., & Mack, I. (2022). Gastrointestinal complaints in patients with anorexia nervosa in the timecourse of inpatient treatment. Frontiers in psychiatry, 13, 962837. | No data on untreated illness |
| 293 | Di Lodovico, L., Al Tabchi, A., Clarke, J., Mancusi, R. L., Messeca, D., Duriez, P., Hanachi, M., & Gorwood, P. (2024). Trajectories and predictive factors of weight recovery in patients with anorexia nervosa completing treatment. A latent class mixed model approach. European eating disorders review : the journal of the Eating Disorders Association, 32(4), 758–770. | No data on untreated illness |
| 294 | Shepherd, C. B., Boswell, R. G., Genet, J., Oliver-Pyatt, W., Stockert, C., Brumm, R., Riebl, S., & Piel, K. (2023). Weight Restoration and Symptom Remission for Longstanding, Untreated Anorexia Nervosa in a Remote Eating Disorder Treatment Program: A Case Study. Clinical Case Studies, 23(3), 230-249. | Inappropriate study design or population |
| 295 | Sandri, E., Cantín Larumbe, E., Cerdá Olmedo, G., Luciani, G., Mancin, S., Sguanci, M., & Piredda, M. (2024). Anorexia and Bulimia Nervosa in Spanish Middle-Aged Adults: Links to Sociodemographic Factors, Diet, and Lifestyle. Nutrients, 16(16), 2671. | No data on untreated illness |
| 296 | Riva, A., Arienti, G., Zuin, G., Spini, L., Calia, M., Biondi, A., Nacinovich, R., & Cavanna, A. E. (2024). Risk Factors for the Development of Eating Disorders in Adolescents with Early-Onset Inflammatory Bowel Diseases. Nutrients, 16(16), 2675. | No data on untreated illness |
| 297 | A Longitudinal Study of Severe and Enduring Eating Disorders. NCT06752304 | Study protocol |
| 298 | Tone J, Chelius B, Miller YD. A Pilot Evaluation of a Focused Acceptance and Commitment Therapy Program for Individuals With an Eating Disorder Waitlisted for Treatment. Int J Eat Disord. 2025;58(11):2116-2125. doi:10.1002/eat.24517 | No data on untreated illness |
| 299 | Collection of abstracts written by members of the Italian Society for the Study of Eating Disorders. Eat Weight Disord 29 (Suppl 1), 26 (2024). https://doi-org.proxy.bib.uottawa.ca/10.1007/s40519-024-01648-6 | Conference abstract |
| 300 | Lubieniecki G, McGrath I, Sharp G. Barriers and facilitators to treatment in severe and enduring eating disorders: a mixed-methods study of access, engagement, and lived experience. J Eat Disord. 2025;13(1):202. Published 2025 Sep 12. doi:10.1186/s40337-025-01393-w | No data on untreated illness |
| 301 | Forrer F, Rubo M, Meyer AH, Munsch S. Binge-eating adolescent treatment (BEAT) - findings from a pilot study on effects and acceptance of a blended treatment program for youth with loss of control eating. BMC Psychol. 2023;11(1):415. Published 2023 Nov 27. doi:10.1186/s40359-023-01429-3 | No data on untreated illness |
| 302 | Steegers C, Bracké K, van der Harst T, et al. Brain, behavior, cognition, and physical health in first-onset adolescent anorexia nervosa: The BRAVE Study design and cohort profile. Apert Neuro. 2024;4:10.52294/001c.116180. doi:10.52294/001c.116180 | No data on untreated illness |
| 303 | Kim JO, An Z, Kim M, Kim YR. Clinical Characteristics and Personality Traits of Pediatric and Adult Patients With Avoidant/Restrictive Food Intake Disorder and Anorexia Nervosa-Restricting Type. Psychiatry Investig. 2025;22(12):1379-1388. doi:10.30773/pi.2025.0038 | No data on untreated illness |
| 304 | Clinical and Cost-effectiveness of Group Schema Therapy for Complex Eating Disorders: the GST-EAT Study. NCT05812950 | Study protocol |
| 305 | Hicks, A. A., & Levy, C. C. (2024). CULTIVATING SYSTEMIC RESILIENCE OF BLACK ADOLESCENTS WITH DISORDERED EATING: Targeting Belief Systems as a Mechanism for Change. In Cultivating Systemic Resilience in Therapy: Applications and Interventions for Families, Relationships, and Individuals (pp. 131-155). Taylor and Francis. https://doi.org/10.4324/9781003373513-9 | Conference abstract |
| 306 | Linda Booij, Kevin Casey, Zsofia Nemoda, Maegan Harvison, Howard Steiger, DNA METHYLATION IN ANOREXIA NERVOSA: A LONGITUDINAL STUDY ON THE ROLE OF CLINICAL STATUS AND ILLNESS DURATION, European Neuropsychopharmacology,  Volume 99, Supplement 1, 2025, Pages 46-47, ISSN 0924-977X, https://doi.org/10.1016/j.euroneuro.2025.08.550. | Conference abstract |
| 307 | Anderson, J., Nelson, V., & Gutta, C. (2025). Delaying Mental Health Screening After Pre-participation Evaluation Improves Athlete Reporting: 1956. Medicine & Science in Sports & Exercise, 57(10S), 643. https://doi.org/10.1249/01.mss.0001160352.47822.b6 | Conference abstract |
| 308 | Developing Recommendations to Support Therapeutic Alliance in Eating Disorders Inpatient Treatment (ALLIANCE). NCT06961032 | Study protocol |
| 309 | Gawron LP, Garrido-Ribas I, Amaro-Carriba MA, et al. Dimensions of insight in eating disorders: clinical and psychological predictors. Psychiatry Res. 2025;351:116566. doi:10.1016/j.psychres.2025.116566 | No data on untreated illness |
| 310 | Mohite K, McTaggart L, Holloway V8168 Exploring the barriers to accessing treatment for eating disorders in adolescents: a literature reviewArchives of Disease in Childhood 2025;110:A3-A4. | Conference abstract |
| 311 | Schmidt, U., Jackson, I. (2024). Early Intervention for Eating Disorders. In: Robinson, P., Wade, T., Herpertz-Dahlmann, B., Fernandez-Aranda, F., Treasure, J., Wonderlich, S. (eds) Eating Disorders. Springer, Cham. https://doi-org.proxy.bib.uottawa.ca/10.1007/978-3-031-46096-8_54 | No data on untreated illness |
| 312 | Egbert A, Schram S. Early intervention for eating disorders: a call to action for inclusion of minoritized groups. Eat Disord. Published online September 30, 2025. doi:10.1080/10640266.2025.2564947 | No data on untreated illness |
| 313 | Obeid N, Porter J, LaMarre A, et al. FREEDcan: an integrated early intervention for eating disorders care model for community and primary care settings in Canada. Transl Behav Med. 2026;16(1):ibaf092. doi:10.1093/tbm/ibaf092 | No data on untreated illness |
| 314 | Halicki-Asakawa A, Mocci J, Libben M. Mobile App-Delivered Motivational Interviewing for Women on Eating Disorder Treatment Waitlists (MI-Coach: ED): Protocol for an App Development and Pilot Evaluation. JMIR Res Protoc. 2025;14:e66298. Published 2025 Apr 10. doi:10.2196/66298 | Study protocol |
| 315 | Downs J, Kelly C. Improving Understanding, Recognition and Treatment for Men With Anorexia Nervosa. Br J Hosp Med (Lond). 2025;86(6):1-15. doi:10.12968/hmed.2024.0643 | No data on untreated illness |
| 316 | Elwyn R. A lived experience response to the proposed diagnosis of terminal anorexia nervosa: learning from iatrogenic harm, ambivalence and enduring hope. J Eat Disord. 2023;11(1):2. Published 2023 Jan 5. doi:10.1186/s40337-022-00729-0 | Conference abstract |
| 317 | Matthews A, Bruening AB, Aarnio-Peterson CM, Kramer R. Predictors of caregiver burden before starting family-based treatment for adolescent anorexia nervosa and associations with weight gain during treatment. Eat Weight Disord. 2023;28(1):21. Published 2023 Feb 21. doi:10.1007/s40519-023-01553-4 | No data on untreated illness |
| 318 | Jensen HAR, Plenborg RB, Davidsen M, Ekholm O, Thygesen LC. Prevalence and determinants of eating disorder risk behaviours among adult women in Denmark: findings from nationwide cross-sectional health surveys between 2000 and 2023. J Eat Disord. 2025;13(1):112. Published 2025 Jun 16. doi:10.1186/s40337-025-01318-7 | No data on untreated illness |
| 319 | Murray MF, Dougherty EN, Weiss SE, Wildes JE. Prospective associations among nonverbal reasoning abilities, illness duration, and eating disorder symptoms in anorexia nervosa and bulimia nervosa. J Eat Disord. 2026;14(1):48. Published 2026 Jan 14. doi:10.1186/s40337-025-01515-4 | No data on untreated illness |
| 320 | Halicki-Asakawa A, Gerlof J, Mayzes-Kotulla E, Libben M. Supporting Individuals on Eating Disorder Waitlists Through App-Based Motivational Interviewing: A Qualitative Evaluation of a Program-Led Pilot Intervention. Int J Eat Disord. 2026;59(1):111-121. doi:10.1002/eat.24561 | No data on untreated illness |
| 321 | Affaticati LM, Capuzzi E, Zuliani T, et al. The Role of Duration of Untreated Illness (DUI) on the Course and Biochemical Parameters of Female Patients Affected by Anorexia Nervosa. Brain Behav. 2025;15(6):e70584. doi:10.1002/brb3.70584 | No data on untreated illness |
| 322 | Lawson R, Watterson R, Kennedy MA, Bulik CM, Jordan J. Time taken to reach treatment for eating disorders in New Zealand. Australas Psychiatry. 2025;33(3):525-529. doi:10.1177/10398562241298082 | No data on untreated illness |
| 323 | Giel K., Schmidt U. Treating eating disorders across different illness stages. Psychotherapy and Psychosomatics doi: 10.1159/000540400 | Conference abstract |
| 324 | UEG Week 2025 Poster Presentations. United European Gastroenterol J. 2025;13(Suppl 8):S803-S1476. Published 2025 Oct 5. doi:10.1002/ueg2.70036 | Conference abstract |
| 325 | Lavan O, Goldstein A, Bar Eyal A, et al. Waiting list intervention for adolescents with anorexia nervosa. Eat Disord. Published online June 23, 2025. doi:10.1080/10640266.2025.2519900 | No data on untreated illness |
| 326 | Stadler J, Moessner M, Becker K, et al. Why Young People With Eating Disorder Symptoms Do Not Seek Help-Exploring Barriers to Help-Seeking. Int J Eat Disord. 2025;58(11):2147-2156. doi:10.1002/eat.24515 | No data on untreated illness |
| 327 | Allen LK, Austin A, Flynn M, Glennon D, Mountford AV, Brown A, et al. Predictors of Treatment Outcome in an Early Intervention Eating Disorder Sample. International Journal of Eating Disorders. 2026;59(3):574-80. | Duplicated population |
| 328 | Austin A, Flynn M, Richards K, Allen K, Mountford AV, Glennon D, et al. The relationship between duration of untreated symptoms and clinical outcomes in first episode eating disorders. Psychiatry Research Communications. 2025;5(2):100217 | Duplicated population |
| 329 | Bühren K, Schwarte R, Fluck F, Timmesfeld N, Krei M, Egberts K, et al. Comorbid psychiatric disorders in female adolescents with first‐onset anorexia nervosa. European Eating Disorders Review. 2014;22(1):39-44 | Duplicated population |
| 330 | Richards KL, Hyam L, Allen KL, Glennon D, Di Clemente G, Semple A, et al. National roll‐out of early intervention for eating disorders: Process and clinical outcomes from first episode rapid early intervention for eating disorders. Early Intervention in Psychiatry. 2023;17(2):202-11 | Duplicated population |
| 331 | McClelland J, Austin A, Flynn M, Mountford VA, Simic M, Schmidt U. Duration of Untreated Eating Disorder and Prodrome in Young People: Characteristics and Relationship to Early Outcomes. Early Interv Psychiatry. 2026;20(3):e70164. doi:10.1111/eip.70164 | No data on untreated illness |
| 332 | Egbert AH, Irizarry B, Lualdi E, Tortolani CC, Donaldson DL, Goldschmidt AB. A qualitative assessment of provider-perceived barriers to implementing family-based treatment for anorexia nervosa in low-income community settings. J Eat Disord. 2024;12(1):51. Published 2024 Apr 25. doi:10.1186/s40337-024-01008-w | Inappropriate study design or population |
| 333 | Wilkins J, Gallagher L, Allen K, Schmidt U. "We're on the journey now together": a qualitative examination of "close others'" perspectives on barriers and facilitators to help-seeking for eating disorders. J Eat Disord. 2026;14(1):157. Published 2026 May 14. doi:10.1186/s40337-026-01626-6 | No data on untreated illness |
| 334 | Werle D, Sablottny L, Tuschen-Caffier B, Svaldi J. Effects of cognitive load on food approach bias in individuals with binge eating disorder. Appetite. 2026;219:108434. doi:10.1016/j.appet.2025.108434 | No data on untreated illness |
| 335 | Welch R, Rao T, Karakus M, Scott E, Doreson A, Ghose SS. State-Level Analysis of Access to Intensive Eating Disorder Care for Medicaid Beneficiaries. Psychiatr Serv. 2026;77(3):209-216. doi:10.1176/appi.ps.20250233 | Inappropriate study design or population |
| 336 | Lee J, Barnett P, Goldsmith LP, et al. Early interventions for first onset of symptoms of mental health conditions: an umbrella review of systematic reviews. BMC Med. 2026;24(1):116. Published 2026 Jan 29. doi:10.1186/s12916-026-04617-1 | Inappropriate study design or population |
| 337 | Mayer G, Lemmer D, von Koenigsmarck BG, Friederich HC, Bauer S. Development and evaluation of a web-based toolkit to inform mental health professionals about digital mental health interventions for eating disorders. J Eat Disord. 2026;14(1):33. Published 2026 Jan 9. doi:10.1186/s40337-025-01518-1 | No data on untreated illness |
| 338 | Arbit SE, Kirsch AC, Nowakowski L, et al. Eating disorder treatment programs compared to controls for adolescents and young adults: Systematic review and three-level meta-analyses. Eat Behav. 2026;60:102070. doi:10.1016/j.eatbeh.2026.102070 | Inappropriate study design or population |
| 339 | Öğütlü H, Gözükara ÖP, Genç A, et al. Presentations of eating disorders among youth in Türkiye before and after COVID-19: lessons learned for the future. J Eat Disord. 2025;13(1):292. Published 2025 Nov 22. doi:10.1186/s40337-025-01475-9 | No data on untreated illness |
| 340 | Resmark G, Giel KE, Ziser K, et al. Informationsbedarf zu Essstörungen bei Betroffenen und ihren Bezugspersonen – Ergebnisse einer bundesweiten Multi-Method-Studie [Need for information on eating disorders among those affected and their caregivers-results of a nationwide multi-method study]. Bundesgesundheitsblatt Gesundheitsforschung Gesundheitsschutz. 2025;68(5):539-547. doi:10.1007/s00103-025-04044-2 | No data on untreated illness |
| 341 | Storto ME, Bailey-Straebler SM, Susser LC. Exploring the Relationship Between Eating Disorders and Reproductive Health, with a Focus on Fertility, Obstetric, and Fetal Outcomes: A Narrative Review. Curr Nutr Rep. 2025;14(1):51. Published 2025 Mar 25. doi:10.1007/s13668-025-00641-4 | Inappropriate study design or population |
| 342 | Sacco R, Camilleri N, Eberhardt J, Umla-Runge K, Newbury-Birch D. A systematic review and meta-analysis on the prevalence of mental disorders among children and adolescents in Europe. Eur Child Adolesc Psychiatry. 2024;33(9):2877-2894. doi:10.1007/s00787-022-02131-2 | Inappropriate study design or population |
| 343 | Holgersen G, Abdi-Dezfuli SE, Friis Darrud S, Stornes Espeset EM, Bircow Elgen I, Nordgreen T. Adolescents' perspectives on a novel digital treatment targeting eating disorders: a qualitative study. BMC Psychiatry. 2024;24(1):423. Published 2024 Jun 5. doi:10.1186/s12888-024-05866-1 | No data on untreated illness |
| 344 | He Q, Zheng H, Zhang J, et al. Association between the frontoparietal network, clinical symptoms and treatment response in individuals with untreated anorexia nervosa. Gen Psychiatr. 2024;37(3):e101389. Published 2024 May 27. doi:10.1136/gpsych-2023-101389 | No data on untreated illness |
| 345 | Coffino JA, Udo T, Grilo CM. Rates of Help-Seeking in US Adults With Lifetime DSM-5 Eating Disorders: Prevalence Across Diagnoses and Differences by Sex and Ethnicity/Race. Mayo Clin Proc. 2019;94(8):1415-1426. doi:10.1016/j.mayocp.2019.02.030 | No data on untreated illness |
| 346 | Colizzi M, Comacchio C, Garzitto M, et al. Clinical heterogeneity of feeding and eating disorders: using personality psychopathology to differentiate "simplex" and "complex" phenotypes. BMC Psychiatry. 2024;24(1):888. Published 2024 Dec 4. doi:10.1186/s12888-024-06345-3 | No data on untreated illness |
| 347 | Wild B, Friederich HC, Gross G, et al. The ANTOP study: focal psychodynamic psychotherapy, cognitive-behavioural therapy, and treatment-as-usual in outpatients with anorexia nervosa--a randomized controlled trial. Trials. 2009;10:23. Published 2009 Apr 23. doi:10.1186/1745-6215-10-23 | Study protocol |
| 348 | Strober, M., Freeman, R. and Morrell, W. (1997), The long-term course of severe anorexia nervosa in adolescents: Survival analysis of recovery, relapse, and outcome predictors over 10–15 years in a prospective study. Int. J. Eat. Disord., 22: 339-360 | Inappropriate study design or population |
| 349 | Zipfel S, Wild B, Groß G, et al. Focal psychodynamic therapy, cognitive behaviour therapy, and optimised treatment as usual in outpatients with anorexia nervosa (ANTOP study): randomised controlled trial. Lancet. 2014;383(9912):127-137. doi:10.1016/S0140-6736(13)61746-8 | No data on untreated illness |
| 350 | Forman SF, McKenzie N, Hehn R, et al. Predictors of outcome at 1 year in adolescents with DSM-5 restrictive eating disorders: report of the national eating disorders quality improvement collaborative. J Adolesc Health. 2014;55(6):750-756. doi:10.1016/j.jadohealth.2014.06.014 | Inappropriate study design or population |
| 351 | Fisher CA, Skocic S, Rutherford KA, Hetrick SE. Family therapy approaches for anorexia nervosa. Cochrane Database Syst Rev. 2019;5(5):CD004780. Published 2019 May 1. doi:10.1002/14651858.CD004780.pub4 | Inappropriate study design or population |
| 352 | Kaye WH, Bulik CM, Thornton L, Barbarich N, Masters K. Comorbidity of anxiety disorders with anorexia and bulimia nervosa. Am J Psychiatry. 2004;161(12):2215-2221. doi:10.1176/appi.ajp.161.12.2215 | No data on untreated illness |
| 353 | Davidson AR, Braham S, Dasey L, Reidlinger DP. Physicians' perspectives on the treatment of patients with eating disorders in the acute setting. J Eat Disord. 2019;7:1. Published 2019 Jan 10. doi:10.1186/s40337-018-0231-1 | No data on untreated illness |
| 354 | Strober, M. and Johnson, C. (2012), The need for complex ideas in anorexia nervosa: Why biology, environment, and psyche all matter, why therapists make mistakes, and why clinical benchmarks are needed for managing weight correction†. Int. J. Eat. Disord., 45: 155-178. | No data on untreated illness |
| 355 | Strober, M., Freeman, R. and Morrell, W. (1999), Atypical anorexia nervosa: Separation from typical cases in course and outcome in a long-term prospective study. Int. J. Eat. Disord., 25: 135-142. | No data on untreated illness |
| 356 | Strober, M. (1981), The significance of bulimia in juvenile anorexia nervosa: An exploration of possible etiologic factors. Int. J. Eat. Disord., 1: 28-43. | Inappropriate study design or population |
| 357 | Stuber, M. and Strober, M. (1987), Group therapy in the treatment of adolescents with bulimia: Some preliminary observations. Int. J. Eat. Disord., 6: 125-131. | No data on untreated illness |
| 358 | Strober, M., Lampert, C., Morrell, W., Burroughs, J. and Jacobs, C. (1990), A controlled family study of anorexia nervosa: Evidence of familial aggregation and lack of shared transmission with affective disorders. Int. J. Eat. Disord., 9: 239-253. | Inappropriate study design or population |
| 359 | Von Holle A, Pinheiro AP, Thornton LM, et al. Temporal patterns of recovery across eating disorder subtypes. Aust N Z J Psychiatry. 2008;42(2):108-117. doi:10.1080/00048670701787610 | Inappropriate study design or population |
| 360 | Reba, L., Thornton, L., Tozzi, F., Klump, K.L., Brandt, H., Crawford, S., Crow, S., Fichter, M.M., Halmi, K.A., Johnson, C., Kaplan, A.S., Keel, P., LaVia, M., Mitchell, J., Strober, M., Woodside, D.B., Rotondo, A., Berrettini, W.H., Kaye, W.H. and Bulik, C.M. (2005), Relationships between features associated with vomiting in purging-type eating disorders. Int. J. Eat. Disord., 38: 287-294. | Inappropriate study design or population |
| 361 | Herzog, D.B., Keller, M.B., Zucker, M.E., Strober, M., Yeh, C. and Pai, S.-Y. (1992), The current status of treatment for anorexia nervosa and bulimia nervosa. Int. J. Eat. Disord., 12: 215-220. | Inappropriate study design or population |
| 362 | Strober, M., Freeman, R., Lampert, C., Diamond, J. and Kaye, W. (2001), Males with anorexia nervosa: A controlled study of eating disorders in first-degree relatives. Int. J. Eat. Disord., 29: 263-269. | No data on untreated illness |

**Supplementary Table 2**: Inclusion and exclusion criteria of included studies

| **Authors** | **Study inclusion criteria** |
| --- | --- |
| Brown et al. (2016) | Patients aged 18–25 years, a primary DSM-5 ED diagnosis and an ED illness duration of ≤3 years. |
| Buhren et al. (2013) | Inclusion criteria were female sex, age between 11-18 and a diagnosis of AN according to DSM-IV criteria. The weight threshold for entering study was a BMI below the 10th percentile. Only patients with first episode who had not received any prior inpatient treatment were included. Exclusion criteria were history if BN or BED, organic brain disease, schizophrenia, bipolar disorder or substance dependence or abuse. |
| Caldiroli et al. (2024) | Inclusion criteria were: (1) age 17–60 years; (2) fluency in Italian language; (3) ability and willingness to sign the written informed consent; (4) diagnosis of AN, BN, BED, or OSFED according to the DSM-5 criteria [1]. Exclusion criteria were: (1) pregnancy or breastfeeding; (2) intellectual disability; (3) presence of a severe organic disease that could affect eating attitudes and weight (e.g., neoplasia-induced cachexia, endocrine imbalance). |
| Campbell et al. (2022) | All family-based treatment referrals of youth under 18 years-old. |
| Castañeda et al. (2024) | At least 14 years old, diagnosed with an eating disorder according to DSM-5, having started treatment for the first time within the last two years, and with at least five or more outpatient treatment sessions, or seven days of inpatient  treatment. Exclusion criteria were severe psychiatric or somatic symptoms that may affect participation, having a previous treatment for the ED, and active drug use. |
| Chew et al. (2020) | NR - consecutive patients in an eating disorder clinic. |
| De Stefani et al. (2023) | The study included all patients who had been treated at a specialised public eating disorder service in the interior of São Paulo, Brazil, from the time the service was established in 1982 until December 2019. Patients of any sex and age were eligible if they had received a clinical diagnosis, via interview, of AN, BN, or EDNOS.  Patients were excluded if they were still in treatment at the time of data collection (July 2020 to January 2021), if they had a different diagnostic hypothesis from those listed above, or if they had not completed at least one month of follow-up within the service. |
| Del Berrio et al. (2022) | Inclusion criteria were being between 15-25 years old when they were in the first 3 years of an ED. |
| Flynn et al. (2020) | Patients aged 16–25, had a primary DSM‐5 ED diagnosis and an ED illness duration of ≤3 years. |
| Gallagher et al. (2025) | NR |
| Guerrero et al. (2022) | No history or specific ED treatment before the inclusion, less than three years of DUED and less than 30 years old. Inclusion criteria for the HC group were no history or current of any psychiatric or physical disorder as well as ED, less than 30 years old and no current use of psychotropic medication, drugs or alcohol addiction. |
| Gumz et al. (2018) | Inclusion: female gender, current diagnosis of AN, participation in diagnostic telephone interview, currently undergoing first eating disorder-specific treatment, provision of written informed consent Exclusion: insufficient German language skills, severe organic or psychological complaints preventing participation (based on recruiting doctor of psychotherapist point of view), ""not living in Hamburg"" an exclusion because they were regarded as not having received the public health intervention |
| Gumz et al. (2023) | 14 or older; female gender; full or sub threshold AN according to DSM-V; first psychotherapeutic treatment; exclusion was not speaking German |
| Harada et al. (2020) | Inclusion criteria were as follows: (a) female; (b) diagnosed with AN-R according to DSM-5, and (c) Japanese speaker |
| Herpertz-Dahlmann et al. (2014) | Female patients were eligible for inclusion if they were aged 11–18 years, had a diagnosis of anorexia nervosa according to Diagnostic and Statistical Manual of Mental Disorders IV (DSM-IV), a body-mass index (BMI) below the tenth percentile (in accordance with international definitions of adolescent anorexia nervosa), and it was their first hospital admission for anorexia nervosa. The decision to admit a patient to the hospital was independent of and made before inclusion into the study and depended on factors in addition to BMI. To make DP treatment feasible, only patients living within a 60 min commute of the treating department were included in the study. Exclusion criteria were as follows: organic brain disease, psychotic or bipolar disorder, substance dependence or abuse, serious self-injurious behaviour, insufficient knowledge of the German language, or an IQ below 85. |
| Jaite et al. (2019) | Children and adolescents up to 18 years diagnosed with AN (according to DSM-5 criteria) by a child and adolescent psychiatrist and first admission to impatient treatment because of AN. |
| Kastner et al. (2021) | NR |
| Kwok et al. (2019) | Patients ≤18 years diagnosed with AN using DSM-IV-TR; excluded if ED-like symptoms due to another primary psychiatric diagnosis |
| Lieberman et al. (2019) | Children aged 8–13 meeting DSM-5 criteria for AN or ARFID; excluded if other ED or no ED diagnosis |
| McClelland et al. (2020) | Patients seen in the Maudsley Centre for Child and Adolescent Eating Disorders who were aged >13 years in 2009, >14 years in 2010, 15 years in 2011, 16 years in 2012, and >17 years in 2013. |
| Monteleone et al. (2023) | Diagnosis of ED according to DSM-V criteria; acceptance to join the study; exclusion was referral to one of the participating ED centres directly from another ED specialist centre |
| Neubauer et al. (2014) | Inclusion criteria were female gender, age between 10 and 60, current diagnosis of AN or subsyndromal AN, according to DSM-IV and ICD-10 criteria, participation in a diagnostic interview, onset of AN in 1990 or later, currently undergoing treatment for AN. Exclusion criteria were insufficient language skills and acute suicidal tendencies. |
| Ng et al. (2018) | NR |
| Reuter et al. (2021) | Inclusion criteria were an age >_ 14 years, female gender and typical or atypical AN diagnosis. We included patients who were either currently in their first AN treatment or who initiated their first psychotherapeutic AN treatment within the last 12 months. |
| Rosello et al. (2021) | Participants were recruited from cases newly presenting to the Surrey Child and Adolescent ED service. The sample included caregivers and their children with ICD-10 ED diagnosis, and who had not been previously diagnosed or treated for an ED. |
| Salas et al. (2011) | Inclusion Criteria: 1. Male participants. 2. Age up to 20 years old (patients treated by the Eating Disorders Unit of the Department of Pediatrics at Pontificia Universidad Católica de Chile, which focuses on children and adolescents). 3.Diagnosis of eating disorders (ED) based on DSM-IV-TR criteria, excluding the amenorrhea criterion for AN.  Exclusion Criteria: Participants with pre-existing major psychiatric conditions such as schizophrenia or psychosis were excluded. |
| Schlegl et al. (2019) | Inclusion criteria included a minimum age of 14 years and a diagnosis of AN (F50.0, F50.1) or BN (F50.2, F50.3) |
| Valenta et al. (2025) | Participants were selected based on information from their medical records, using the following inclusion criteria: (a) a diagnosis of EDs according to DSM-5 criteria, (b) age over 18 years at the time of assessment, (c) adequate proficiency in Italian, and (d) provision of informed consent to participate in the study and consent for the use of their data for research purposes. Exclusion criteria were poor understanding  of Italian language, ongoing psychotic disorders, or cognitive deficits. |
| Weigel et al. (2014) | Inclusion criteria: Female gender; Aged between 10 and 60 years; Primary diagnosis of anorexia nervosa (AN) or subsyndromal AN (one diagnostic criterion absent) based on DSM-IV; Current treatment for AN (first treatment for AN); In- or outpatient treatment in a specialized treatment unit; Informed consent provided (with additional consent from parents/guardians for participants under 16 years).  Exclusion criteria: Insufficient language skills; Severe organic or psychiatric comorbidity; Acute suicidal intentions. |

**Supplementary Figure 1**: Funnel plot of total pooled results standard errors versus duration of untreated illness

**Supplementary Figure 2**: Scatter plot showing meta-regression for mean age against duration of untreated illness of total pooled results. Blue line represents the regression line.

**Supplementary Figure 3:** Forest plot showing duration of untreated illness in people with anorexia nervosa

**Supplementary Figure 4**: Funnel plot of pooled results standard errors versus duration of untreated illness in patients with anorexia nervosa

**Supplementary Figure 5**: Scatter plot showing meta-regression for mean age against duration of untreated illness in the anorexia nervosa subgroup. Blue line represents the regression line.

**Supplementary Figure 6**: Forest plot showing duration of untreated illness in the mixed-diagnosis subgroup

**Supplementary Figure 7**: Funnel plot of pooled results standard errors versus duration of untreated illness in studies with mixed eating disorders diagnosis

**Supplementary Figure 8**: Scatter plot showing meta-regression for mean age against duration of untreated illness in the in the mixed-diagnosis subgroup. Blue line represents the regression line.

**Supplementary Table 3**: Sensitivity analyses showing meta-analysis results with one-study removed

| **Study removed** | **DUED (months)** | **lower**  **95% CI** | **higher 95% CI** | **I^2^** |
| --- | --- | --- | --- | --- |
| Chew et al (2021) | 22.23 | 18.31 | 26.14 | 99.76 |
| Gumz et al (2023) | 22.23 | 18.31 | 26.14 | 99.77 |
| Del Barrio et al (2021) | 22.22 | 18.3 | 26.14 | 99.77 |
| Reuter et al (2021) | 22.21 | 18.29 | 26.13 | 99.78 |
| Campbell et al (2022) | 22.21 | 18.29 | 26.14 | 99.76 |
| Lieberman et al (2019) | 22.21 | 18.28 | 26.13 | 99.77 |
| Del Barrio et al (2021) | 22.2 | 18.27 | 26.13 | 99.77 |
| Jaite et al (2019) | 22.2 | 18.27 | 26.13 | 99.77 |
| Guerrero et al (2022) | 22.16 | 18.22 | 26.1 | 99.78 |
| Del Barrio et al (2021) | 22.16 | 18.22 | 26.11 | 99.78 |
| Chew et al (2021) | 22.16 | 18.22 | 26.1 | 99.77 |
| Del Barrio et al (2021) | 22.16 | 18.22 | 26.1 | 99.77 |
| Salas et al (2011) | 22.16 | 18.21 | 26.1 | 99.78 |
| Rosello et al (2020) | 22.15 | 18.21 | 26.1 | 99.78 |
| Jaite et al (2019) | 22.15 | 18.2 | 26.09 | 99.77 |
| Buhren et al (2013) | 22.14 | 18.19 | 26.09 | 99.78 |
| Herpertz-Dahlmann et al (2014) | 22.13 | 18.18 | 26.08 | 99.77 |
| Guerrero et al (2022) | 22.11 | 18.15 | 26.06 | 99.78 |
| Rosello et al (2020) | 22.1 | 18.15 | 26.06 | 99.78 |
| Rosello et al (2020) | 22.09 | 18.13 | 26.05 | 99.78 |
| Harada et al (2020) | 22.07 | 18.11 | 26.03 | 99.78 |
| Gallagher et al (2025) | 22.07 | 18.11 | 26.04 | 99.71 |
| Guerrero et al (2022) | 22.05 | 18.1 | 26.01 | 99.78 |
| Gallagher et al (2025) | 22.06 | 18.09 | 26.02 | 99.78 |
| Brown et al (2016) | 22.04 | 18.07 | 26 | 99.78 |
| Gallagher et al (2025) | 22.03 | 18.06 | 26 | 99.64 |
| Gumz et al (2023) | 22.02 | 18.05 | 25.99 | 99.78 |
| Flynn et al (2020) | 22.01 | 18.04 | 25.98 | 99.78 |
| Brown et al (2016) | 21.99 | 18.02 | 25.96 | 99.78 |
| Flynn et al (2020) | 21.97 | 18 | 25.94 | 99.78 |
| McClelland et al (2020) | 21.91 | 17.96 | 25.85 | 99.78 |
| Monteleone et al (2023) | 21.89 | 17.92 | 25.86 | 99.77 |
| Neubauer et al. (2014) | 21.87 | 17.9 | 25.83 | 99.78 |
| Harada et al (2020) | 21.86 | 17.9 | 25.83 | 99.78 |
| McClelland et al (2020) | 21.86 | 17.91 | 25.81 | 99.78 |
| McClelland et al (2020) | 21.86 | 17.91 | 25.81 | 99.78 |
| Valenta et al (2025) | 21.84 | 17.88 | 25.8 | 99.78 |
| Ng et al (2018) | 21.83 | 17.87 | 25.79 | 99.78 |
| Lieberman et al (2019) | 21.8 | 17.86 | 25.74 | 99.78 |
| Caldiroli et al (2024) | 21.78 | 17.84 | 25.73 | 99.78 |
| McClelland et al (2020) | 21.85 | 17.94 | 25.76 | 99.78 |
| Kwok et al (2019) | 21.72 | 17.78 | 25.67 | 99.78 |
| McClelland et al (2020) | 21.78 | 17.85 | 25.71 | 99.78 |
| Weigel et al (2014) | 21.78 | 17.85 | 25.71 | 99.78 |
| Harada et al (2020) | 21.71 | 17.77 | 25.65 | 99.78 |
| McClelland et al (2020) | 21.7 | 17.77 | 25.64 | 99.78 |
| Reuter et al (2021) | 21.7 | 17.78 | 25.62 | 99.78 |
| Gumz et al (2018) | 21.69 | 17.77 | 25.61 | 99.78 |
| Kastner et al (2021) | 21.85 | 17.95 | 25.75 | 99.78 |
| Schlegl et al. (2019) | 21.54 | 17.65 | 25.43 | 99.77 |
| Gumz et al (2018) | 21.79 | 17.89 | 25.69 | 99.78 |
| De Stefani et al (2023) | 21.31 | 17.51 | 25.11 | 99.76 |
| Ng et al (2018) | 21.44 | 17.61 | 25.28 | 99.77 |
| Valenta et al (2025) | 21.16 | 17.43 | 24.89 | 99.75 |
| Kwok et al (2019) | 21.39 | 17.58 | 25.2 | 99.76 |
| Ng et al (2018) | 21.17 | 17.44 | 24.9 | 99.75 |
| Schlegl et al. (2019) | 21.06 | 17.38 | 24.74 | 99.74 |
| Castaneda et al (2024) | 21.26 | 17.52 | 25 | 99.76 |

**Supplementary Table 4**: Sensitivity analyses showing meta-analysis results with one-study removed in AN sub-group analysis

| **Study removed** | **DUED (months)** | **lower**  **95% CI** | **higher 95% CI** | **I^2^** |
| --- | --- | --- | --- | --- |
| Gumz et al (2023) | 20.71 | 15.18 | 26.25 | 99.14 |
| Reuter et al (2021) | 20.68 | 15.11 | 26.24 | 99.23 |
| Lieberman et al (2019) | 20.67 | 15.09 | 26.24 | 99.09 |
| Del Barrio et al (2021) | 20.65 | 15.06 | 26.24 | 99.15 |
| Jaite et al (2019) | 20.64 | 15.05 | 26.24 | 99.09 |
| Guerrero et al (2022) | 20.56 | 14.91 | 26.2 | 99.25 |
| Jaite et al (2019) | 20.52 | 14.85 | 26.19 | 99.14 |
| Buhren et al (2013) | 20.49 | 14.8 | 26.17 | 99.2 |
| Herpertz-Dahlmann et al (2014) | 20.48 | 14.78 | 26.17 | 99.18 |
| Harada et al (2020) | 20.32 | 14.58 | 26.05 | 99.28 |
| Gumz et al (2023) | 20.18 | 14.44 | 25.92 | 99.29 |
| Neubauer et al. (2014) | 19.74 | 14.05 | 25.44 | 99.28 |
| Harada et al (2020) | 19.73 | 14.03 | 25.43 | 99.27 |
| Ng et al (2018) | 19.64 | 13.98 | 25.31 | 99.27 |
| Kwok et al (2019) | 19.31 | 13.76 | 24.86 | 99.22 |
| Weigel et al (2014) | 19.56 | 14.02 | 25.11 | 99.26 |
| Harada et al (2020) | 19.28 | 13.76 | 24.81 | 99.23 |
| Reuter et al (2021) | 19.35 | 13.86 | 24.83 | 99.24 |
| Gumz et al (2018) | 19.33 | 13.86 | 24.8 | 99.23 |
| Kastner et al (2021) | 19.82 | 14.35 | 25.29 | 99.25 |
| Schlegl et al. (2019) | 18.74 | 13.52 | 23.96 | 99.13 |
| Gumz et al (2018) | 19.67 | 14.2 | 25.14 | 99.25 |
| Kwok et al (2019) | 18.63 | 13.58 | 23.69 | 99.1 |

**Supplementary Table 5**: Sensitivity analyses showing meta-analysis results with one-study removed in the mixed eating disorders diagnosis sub-group analysis

| **Study removed** | **DUED (months)** | **lower**  **95% CI** | **higher 95% CI** | **I^2^** |
| --- | --- | --- | --- | --- |
| Chew et al (2021) | 21.6 | 16.7 | 26.5 | 99.78 |
| Campbell et al (2022) | 21.57 | 16.64 | 26.5 | 99.78 |
| Chew et al (2021) | 21.47 | 16.46 | 26.47 | 99.8 |
| Salas et al (2011) | 21.46 | 16.45 | 26.46 | 99.81 |
| Rosello et al (2020) | 21.45 | 16.44 | 26.47 | 99.81 |
| Guerrero et al (2022) | 21.36 | 16.31 | 26.41 | 99.81 |
| Rosello et al (2020) | 21.35 | 16.29 | 26.41 | 99.81 |
| Rosello et al (2020) | 21.33 | 16.26 | 26.39 | 99.81 |
| Gallagher et al (2025) | 21.29 | 16.21 | 26.37 | 99.7 |
| Guerrero et al (2022) | 21.24 | 16.18 | 26.3 | 99.82 |
| Gallagher et al (2025) | 21.25 | 16.16 | 26.34 | 99.81 |
| Brown et al (2016) | 21.21 | 16.12 | 26.3 | 99.82 |
| Gallagher et al (2025) | 21.2 | 16.1 | 26.3 | 99.63 |
| Flynn et al (2020) | 21.16 | 16.05 | 26.26 | 99.81 |
| Brown et al (2016) | 21.1 | 16 | 26.21 | 99.82 |
| Flynn et al (2020) | 21.07 | 15.96 | 26.17 | 99.81 |
| McClelland et al (2020) | 20.94 | 15.93 | 25.95 | 99.81 |
| Monteleone et al (2023) | 20.89 | 15.8 | 25.98 | 99.8 |
| McClelland et al (2020) | 20.84 | 15.81 | 25.88 | 99.81 |
| McClelland et al (2020) | 20.85 | 15.83 | 25.86 | 99.81 |
| Valenta et al (2025) | 20.79 | 15.73 | 25.85 | 99.81 |
| Caldiroli et al (2024) | 20.68 | 15.69 | 25.68 | 99.81 |
| McClelland et al (2020) | 20.86 | 15.95 | 25.78 | 99.81 |
| McClelland et al (2020) | 20.71 | 15.75 | 25.67 | 99.81 |
| McClelland et al (2020) | 20.49 | 15.54 | 25.44 | 99.81 |
| De Stefani et al (2023) | 19.56 | 15.17 | 23.94 | 99.75 |
| Valenta et al (2025) | 19.14 | 15.08 | 23.21 | 99.7 |
| Castaneda et al (2024) | 19.88 | 15.37 | 24.38 | 99.77 |

 **Supplementary Figure 9:** Influence diagnostics for the meta-analysis of duration of untreated eating disorder (DUED).
